# Nanomedicines Targeting Metabolic Pathways in the Tumor Microenvironment: Future Perspectives and the Role of AI

**DOI:** 10.3390/metabo15030201

**Published:** 2025-03-13

**Authors:** Shuai Fan, Wenyu Wang, Wenbo Che, Yicheng Xu, Chuan Jin, Lei Dong, Qin Xia

**Affiliations:** State Key Laboratory of Molecular Medicine and Biological Diagnosis and Treatment (Ministry of Industry and Information Technology), Aerospace Center Hospital, School of Life Science, Beijing Institute of Technology, Beijing 100081, China; 1120221191@bit.edu.cn (S.F.); 1120223086@bit.edu.cn (W.W.); 1120241580@bit.edu.cn (W.C.); 1120220699@bit.edu.cn (Y.X.); 1120230798@bit.edu.cn (C.J.)

**Keywords:** nanomedicine, tumor, metabolism, TME, artificial intelligence

## Abstract

**Background:** Tumor cells engage in continuous self-replication by utilizing a large number of resources and capabilities, typically within an aberrant metabolic regulatory network to meet their own demands. This metabolic dysregulation leads to the formation of the tumor microenvironment (TME) in most solid tumors. Nanomedicines, due to their unique physicochemical properties, can achieve passive targeting in certain solid tumors through the enhanced permeability and retention (EPR) effect, or active targeting through deliberate design optimization, resulting in accumulation within the TME. The use of nanomedicines to target critical metabolic pathways in tumors holds significant promise. However, the design of nanomedicines requires the careful selection of relevant drugs and materials, taking into account multiple factors. The traditional trial-and-error process is relatively inefficient. Artificial intelligence (AI) can integrate big data to evaluate the accumulation and delivery efficiency of nanomedicines, thereby assisting in the design of nanodrugs. **Methods:** We have conducted a detailed review of key papers from databases, such as ScienceDirect, Scopus, Wiley, Web of Science, and PubMed, focusing on tumor metabolic reprogramming, the mechanisms of action of nanomedicines, the development of nanomedicines targeting tumor metabolism, and the application of AI in empowering nanomedicines. We have integrated the relevant content to present the current status of research on nanomedicines targeting tumor metabolism and potential future directions in this field. **Results:** Nanomedicines possess excellent TME targeting properties, which can be utilized to disrupt key metabolic pathways in tumor cells, including glycolysis, lipid metabolism, amino acid metabolism, and nucleotide metabolism. This disruption leads to the selective killing of tumor cells and disturbance of the TME. Extensive research has demonstrated that AI-driven methodologies have revolutionized nanomedicine development, while concurrently enabling the precise identification of critical molecular regulators involved in oncogenic metabolic reprogramming pathways, thereby catalyzing transformative innovations in targeted cancer therapeutics. **Conclusions:** The development of nanomedicines targeting tumor metabolic pathways holds great promise. Additionally, AI will accelerate the discovery of metabolism-related targets, empower the design and optimization of nanomedicines, and help minimize their toxicity, thereby providing a new paradigm for future nanomedicine development.

## 1. Introduction

Since Otto Warburg discovered the phenomenon of aerobic glycolysis in tumor cells, an increasing number of researchers have focused on the formation of the tumor microenvironment (TME) and the metabolic reprogramming of tumors [1]. Tumor cells in solid tumors reside in the TME, where their demands for substances and energy differ significantly from those of normal cells. Most metabolic pathways undergo substantial alterations in order to meet the cell’s needs in this highly complex local environment and potentially to achieve immune suppression [2,3]. Abnormal levels of glucose metabolism, lipid metabolism, amino acid metabolism, and nucleotide metabolism are observed in tumors, accompanied by significant alterations in the concentrations of substances and enzyme levels and catalytic activities within various metabolic pathways. These changes are also associated with the remodeling of the physicochemical properties of the entire microenvironment. Therefore, targeting key factors or components in tumor metabolism with drugs has become an important approach in cancer therapy [4]. However, conventional metabolic-targeting drugs face substantial clinical limitations, primarily due to their inadequate targeting efficiency and the risk of systemic toxicity [5,6].

Fortunately, nanomedicine, an interdisciplinary field combining nanobiotechnology and medicine, which aims to leverage nanoscale materials and technologies for disease treatment, offers great promise due to its high payload capacity, encapsulation efficiency, excellent biocompatibility, and superior ability to specifically target delivery to the TME [7,8,9]. Since 1989, approximately 80 nanomedicines have been approved by the U.S. Food and Drug Administration (FDA) and the European Medicines Agency (EMA) for market use [10]. These nanomedicines span various therapeutic areas, including cancer, infections, and inflammation. As an extension of medicine, nanomedicine primarily relies on biocompatible nanoparticles composed of nanomaterials and small drug molecules to achieve disease diagnosis and therapy [11]. Through the precise design and functionalization of nanomaterials, nanomedicine demonstrates immense potential in personalized therapy and disease monitoring [12,13]. This progress underscores the potential of nanomedicines to enhance therapeutic efficacy, improve drug delivery, and reduce side effects, highlighting their significant role in modern healthcare systems [14]. Most importantly, an increasing number of smart, responsive nanoparticle-based drugs have been developed, such as those utilizing nanoparticles that respond to low pH, high concentrations of glutathione (GSH), and specific enzymes in the TME, making targeted and controllable delivery to the TME feasible [15]. These nanoparticle drugs can interfere with specific metabolic pathways or influence certain metabolites through particular modifications or drug encapsulation designs. Furthermore, precise targeted delivery and controlled release enhance their metabolic targeting ability, potentially even reversing the local characteristics of the microenvironment.

The design process of nanomedicines is highly complex, requiring the integration of multiple mechanisms. As research progresses, an increasing number of nanomedicine designs aim to couple multiple functionalities to enhance therapeutic outcomes [16]. The complexity of nanoscale operations adds a layer of difficulty to the clinical translation of nanomedicines and biomedical nanotechnology, further complicating the design and optimization of nanomaterials for biomedical purposes [17]. The focus of drug development lies in the selection of targets. An increasing number of nanoparticle-based drugs are shifting towards targeted inhibition, making efficient target screening and drug design of paramount importance. Drug design must consider delivery efficiency and selectivity, as well as control over drug release, toxicity reduction, and synergistic effects with other therapeutic approaches. Consequently, the development of nanomedicines is increasingly leaning towards multifunctionality to address the demands of complex disease treatments. To achieve rational design and high-throughput development of nanodrug formulations, it is essential to precisely control the physicochemical properties of nanomaterials and accurately predict their interactions in complex biological environments [18]. By fine-tuning parameters, such as the size, morphology, and surface characteristics of nanomaterials, and combining these with advanced computational methods to predict biological interactions, drug release mechanisms, and biodistribution, it is possible to ensure the safety, stability, and efficacy of the formulations in both in vivo and in vitro settings. This enables the efficient development and precise application of nanomedicine drug delivery systems [19].

Artificial intelligence (AI), a branch of computer science, is designed to perform complex tasks requiring “human intelligence” through computers or computer-controlled machines. AI encompasses various subfields, including machine learning (ML), artificial neural networks (ANNs), and deep learning (DL) [20]. AI is increasingly becoming a driving force for transformation in the biopharmaceutical industry, particularly in the development of nanotechnology products [21]. By processing and analyzing vast datasets, AI can assist in the development of nanomedicines targeting specific metabolic pathways and, based on omics data, analyze individualized tumor metabolic pathways and screen new targets that enhance therapeutic efficacy while minimizing side effects [22]. Through pattern recognition and the continuous optimization of algorithms, AI can optimize the design of nanomedicines, predict the delivery efficiency, toxicity, and other properties of nanoparticle drugs, and assist in providing precise treatment for patients.

## 2. An Overview of the Metabolic Reprogramming of Tumor Cells and the Formation of the Tumor Microenvironment

**Figure 1:**
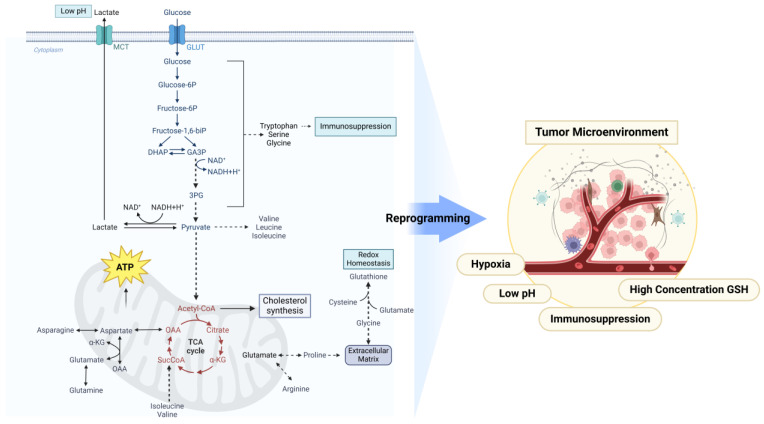
Formation and characteristics of the tumor microenvironment (TME) (dashed lines and multiple arrows indicate omitted multistep reactions). Solid tumors undergo metabolic reprogramming to meet their intrinsic needs, and the features of the TME can be utilized for the development of nanomedicines for controlled drug delivery. The TME is characterized by four main features: (1) hypoxic conditions due to insufficient angiogenesis; (2) increased lactate concentration resulting from upregulated anaerobic glycolysis, leading to a decrease in the pH of the TME; (3) severe immune suppression within the TME caused by various metabolic products; and (4) upregulated GSH levels, which enhance the regulation of redox homeostasis in tumor cells. MCT (Monocarboxylate Transporter), GLUT (Glucose Transporter), DHAP (Dihydroxyacetone Phosphate), GA3P (Glyceraldehyde-3-Phosphate), 3PG (3-Phosphoglycerate), NADH (Nicotinamide Adenine Dinucleotide, Reduced), NAD^+^ (Nicotinamide Adenine Dinucleotide, Oxidized), OAA (Oxaloacetate), α-KG (Alpha-Ketoglutarate), SucCoA (Succinyl-CoA). Created with Biorender.com (accessed on 24 February 2025).

To meet the tumor’s increased demand for nutrients and energy, significant metabolic reprogramming occurs in tumor cells, including alterations in glucose metabolism, lipid metabolism, amino acid metabolism, and nucleotide metabolism. The proliferative rate of tumor cells exceeds the oxygen supply provided by the vasculature, resulting in a hypoxic TME that further promotes the metabolic reprogramming of the tumor [23]. Metabolic reprogramming in solid tumors often leads to elevated concentrations of certain metabolites in the TME, resulting in a decrease in pH, high levels of GSH, and the establishment of immune suppression characteristics. These changes further promote tumorigenesis, tumor progression, and the development of resistance (Figure 1).

### 2.1. Reprogramming of Glucose Metabolism in Cancer

Glucose is the most abundant nutrient in the blood and is basically also the main source of energy in cells [24]. Cancer cells employ upregulated glycolytic pathways for glucose catabolism, a process that generates metabolic precursors and intermediates essential to support diverse biosynthetic pathways [25]. When in cytosol, glucose may be used as a substrate in glycolysis (where the resulting pyruvate contributes to acetyl-CoA synthesis, crucial for the production of fatty acids (FAs), lipids, cholesterol, and nonessential amino acids) [26].

#### 2.1.1. Aerobic Glycolysis in Cancer

Studies have shown that increased glucose uptake in cancer cells is associated with poor prognosis and cancer metastasis [27]. In the initiation, progression, and metastasis of tumors, glucose metabolic enzymes and transport proteins play crucial roles. Otto Warburg elucidated the Warburg effect, characterizing it as a distinct metabolic pattern exhibited by cancer cells, which is typified by a transition from oxidative phosphorylation (OXPHOS) to aerobic glycolysis [28]. The upregulation of glycolysis is a significant change in glucose metabolism in many primary and metastatic cancers, and aerobic glycolysis is considered to be the most effective mechanism in cancer cell glucose metabolism, representing an evolutionary adaptation [29]. The glycolysis process involves a series of intermediate metabolites, converting glucose into pyruvate, accompanied by the generation of ATP and Nicotinamide adenine dinucleotide (NADH).

Aerobic glycolysis, a hallmark of cancer metabolism, is a tightly regulated process that can be divided into two distinct phases. In the initial phase, glucose is metabolized into pyruvate, NADH, and a limited yield of ATP (only two molecules per glucose molecule) [30]. Under normoxic conditions, the energy stored in NADH is further harnessed through the mitochondrial electron transport chain [31]. However, under hypoxic conditions, pyruvate is reduced to lactate, a critical step in the second phase of aerobic glycolysis. This metabolic pathway is characterized by its inefficiency in glucose utilization, necessitating increased glucose uptake to sustain cellular energy demands [32]. Many studies have confirmed that aerobic glycolysis in cancer cells increases even in the presence of oxygen [33].

Phase I: Energy Investment and Metabolic Reprogramming in Cancer. The first phase of glycolysis, often referred to as the energy investment phase, involves the ATP-dependent phosphorylation of glucose, a process known as priming [34]. This step is catalyzed by hexokinase (HK), which converts glucose into glucose-6-phosphate (G6P) [35]. In cancer cells, the isoform HK-2, which localizes to the outer mitochondrial membrane, plays a pivotal role in enhancing glycolytic flux [36]. HK-2 exhibits two critical functional properties: It efficiently utilizes ATP through its interaction with mitochondrial adenine nucleotide translocase (ANT). It is resistant to feedback inhibition by its product, G6P, allowing sustained glycolytic activity [37]. Furthermore, HK-2 interacts with the p53-regulated protein TP53-induced glycolysis and apoptosis regulator (TIGAR) [38]. Under hypoxic conditions, TIGAR forms a complex with HK-2, augmenting its enzymatic activity [39]. Subsequently, p53 interacts with TIGAR and activates TIGAR’s fructose-2,6-bisphosphatase activity, resulting in an enhanced production of NADPH [40]. The metabolic adaptation of cancer cells not only modulates mitochondrial reactive oxygen species (ROS) levels but also promotes cancer cell survival, highlighting the intricate interplay between metabolic reprogramming and tumor progression [41].

Phase II: Energy Payoff and the Warburg Effect. The second phase of glycolysis, termed the energy payoff phase, encompasses a series of enzymatic reactions that further catabolize G6P into pyruvate, generating ATP and NADH [42]. The end product, pyruvate, can either enter the mitochondria to participate in the tricarboxylic acid (TCA) cycle or be reduced to lactate under hypoxic conditions. In cancer cells, glucose is used to produce lactate in the presence of oxygen through a process termed aerobic glycolysis [43]. This metabolic reprogramming, epitomized by the Warburg Effect, enables cancer cells to preferentially utilize glycolysis for energy production, even in the presence of adequate oxygen [44]. This metabolic shift not only supplies the ATP and biosynthetic precursors necessary for uncontrolled cell growth but also confers a survival advantage in the hypoxic TME [45]. The production and secretion of lactate acidify the extracellular milieu, which can suppress antitumor immune responses and promote tumor invasion, thereby actively shaping the TME to facilitate cancer progression [46].

#### 2.1.2. Key Catalytic Enzymes in Tumor Aerobic Glycolysis

Phosphoglucose isomerase (PGI) is a protein with multiple functions that plays different roles inside and outside the cell. As a housekeeping enzyme within the cell, it acts as a cytokine outside the cell [47]. The catalytic action of PGI is crucial in both glycolysis and gluconeogenesis, responsible for catalyzing the interconversion of G6P and F6P [48]. The expression level of PGI is elevated in cancer and its metastasis, and the role of PGI as a cytokine in cancer is closely related to its interaction with its receptors [49].

Phosphofructokinase (PFK) plays a crucial role in the glycolysis process by irreversibly catalyzing the phosphorylation of F6P to produce Fructose-1,6-bisphosphate (FBP), thereby regulating the second key step of this metabolic pathway. Phosphofructokinase-1 (PFK1) is the main rate-limiting enzyme in glycolysis that converts F6P into FBP [50]. PFK1 can be allosterically activated by the metabolic product of 6-phosphofructo-2-kinase/fructose-2,6-bisphosphatase 4 (PFKFB4)—fructose-2,6-bisphosphate. Studies have shown that PFKFB4 can directly phosphorylate steroid receptor coactivator-3 (SRC-3) and enhance its transcriptional activity, thereby promoting tumor metastasis [51]. Phosphofructokinase-2 (PFK2) expression is significantly increased in various invasive primary tumors [52]. PFK2 acts as a crucial regulator of glycolysis under hypoxic conditions, and its role is considered essential [53].

Fructose-bisphosphate aldolase, commonly known as “aldolase (ALDO)”, is an enzyme responsible for cleaving fructose 1,6-bisphosphate into dihydroxyacetone phosphate (DHAP) and glyceraldehyde 3-phosphate (G3P) [54]. The nuclear isoform of aldolase A significantly contributes to cellular division by engaging in interactions with F-actin and members of the WASP family, which are pivotal in regulating the polymerization of actin filaments [55]. Aldolase B suppresses hepatocellular carcinogenesis through moonlighting interactions with multiple protein targets, including G6P dehydrogenase [56].

Glyceraldehyde 3-phosphate dehydrogenase (GAPDH), phosphoglycerate kinase (PGK), and phosphoglycerate mutase (PGAM) catalyze three sequential glycolytic steps converting G3P to 2-phosphoglycerate (2-PG) [57]. GAPDH constitutes a pivotal enzyme involved in the regulation of glycolysis and is highly associated with tumors [58,59]. Its increased enzymatic activity can be associated with PKM2 which is a significant characteristic in malignant cells [60]. GAPDH overexpression has been reported in several cancers, such as colon cancer, liver metastasis, melanoma, and prostate cancer [61,62,63,64]. GAPDH antagonists induce apoptosis and inhibit tumor progression and GAPDH inhibition can overcome chemotherapy resistant responses [65].

Phophoglycerate mutase 1 (PGAM1) is a glycolytic enzyme that interconverts 3-phosphoglycerate (3-PG) and 2-PG [66]. PGAM1 orchestrates glycolysis, PPP flux, and macromolecular biosynthesis by controlling the 3-PG/2-PG ratio in rapidly growing tumors [67]. PGAM1 overexpression was reported in several cancers, and it might be associated with tumor growth, survival, and metastasis [68,69]. Enolase interconverts 2-PG to phosphoenolpyruvate in glycolysis. The α-enolase enzyme is encoded by the ENO1 gene, and it plays several roles in tumor invasion and metastasis [70]. ENO1 promotes metastasis and cell migration via regulating the AKT signaling pathway in gastric cancer [71]. It also increases bladder cancer cell growth and proliferation via the β-catenin pathway [72]. PKM2 is the last step-rate-limiting enzyme in the glycolytic process, which converts phosphoenolpyruvate to pyruvate [73]. PKM2 is overexpressed in several types of cancer and it regulates the redox homeostasis in cancer cells [74].

#### 2.1.3. Lactate Metabolism

Lactate is an end product of aerobic glycolysis. As a critical energy source for mitochondrial respiration, lactate also acts as a precursor of gluconeogenesis and a signaling molecule [75]. Lactate is a key player in cancer and metastasis in both the normoxic and hypoxic condition. Furthermore, increased antioxidant features in cancer cells lead to the radioresistance condition [76]. Discharge of lactate into the cells surrounding constructing cells is an essential phenomenon in preparing the favorable niche for cancer growth and this phenomenon is strongly associated with PKM2 [77]. In tumor cells, glycolysis facilitates the conversion of excess pyruvate and NADH into lactate and NAD+ through the action of lactate dehydrogenase A (LDHA), thereby contributing to ATP production to some extent [78]. LDH is a critical enzyme that transforms pyruvate into lactate and it is overexpressed in various cancer cells [79]. The high utilization of glucose by cancer cells results in the accumulation of extracellular lactate [80]. Due to the significant accumulation of lactic acid, the extracellular pH value in the TME becomes acidic. This acidic environment promotes processes, such as metastasis and angiogenesis, and more importantly, it is associated with immunosuppression, which has been proven to be related to poorer clinical outcomes [81].

Lactate, together with other metabolic intermediates, fuels the TCA cycle [82]. The TCA cycle oxidizes acetyl-CoA derived from carbohydrates, lipids, and proteins, ultimately generating carbon dioxide and NADH, among other products [83]. In the TCA cycle, succinate dehydrogenase (SDH), isocitrate dehydrogenase (IDH), and fumarate hydratase (FH) are classified as tumor suppressors [84].

#### 2.1.4. Pentose Phosphate Pathway and Gluconeogenesis

The pentose phosphate pathway (PPP) runs alongside glycolysis, diverging from G6P to produce NADPH and ribose 5-phosphate (R5P) [85]. The PPP is crucial for supporting glycolytic cancer cells in fulfilling their anabolic needs and managing oxidative stress, as NADPH is essential for fatty acid synthesis and the neutralization of reactive oxygen species (ROS), both of which consume NADPH [86]. In addition, the increased glycolytic flux in cancer could indirectly affect the PPP. These regulations are vital for the survival and proliferation of tumor cells [87].

Gluconeogenesis is a series of enzymatic reactions, which utilize noncarbohydrate substrates to generate glucose [88]. The majority of gluconeogenesis reactions are the reverse of glycolytic reactions, except for the irreversible steps. The irreversible reactions in gluconeogenesis are catalyzed by the following enzymes: pyruvate carboxylase, phosphoenolpyruvate carboxykinase 1 (PEPCK1, or PCK1), fructose-1,6-bisphosphatase 1 and 2, and glucose-6-phosphatase (G6Pase) [89]. Gluconeogenesis plays a significant role in suppressing aerobic glycolysis and impacts various other metabolic pathways in cancer cells, such as the TCA cycle, OXPHOS, the PPP, glutaminolysis, as well as serine and nucleotide biosynthesis [90]. Certain cancer types exploit key gluconeogenic enzymes to initiate truncated gluconeogenesis, thereby promoting metabolic flexibility. This mechanism enables cells to utilize non-carbohydrate precursors for biosynthesis and redirects the flux of glucose metabolism toward the production of antioxidants [91].

### 2.2. Reprogramming of Lipid Metabolism in Cancer

Among cancer cells’ metabolic alterations, the dysregulation of lipid metabolism is one of the most prominent features [92]. Cancer cells exploit lipid metabolism to fuel their proliferation, invasion, metastasis, and adaptation to the TME [93]. Additionally, lipid metabolism provides essential energy, biomembrane components, and signaling molecules, which are critical for cancer progression and resistance to therapeutic interventions [94].

#### 2.2.1. The Central Role of Fatty Acids in Cancer Cell Proliferation

Cell proliferation relies heavily on FAs for the synthesis of cellular membranes and signaling molecules [95,96]. Structurally, FAs consist of a carboxyl group and a hydrocarbon chain, typically containing an even number of carbon atoms [97]. These molecules play pivotal roles in energy storage, membrane biogenesis, and the generation of signaling lipids [98]. FAs are fundamental components of glycolipids and phospholipids, which are essential for maintaining cellular structure and function [99]. Under nutrient-rich conditions, FAs esterify with glycerol to form triglycerides, which are stored in lipid droplets (LDs) as energy reserves. Lipid droplet accumulation has been observed in many cancers and is elevated in cancer cells exposed to hypoxia or nutrient starvation [100,101]. During energy stress, these triglycerides are hydrolyzed, releasing FAs that undergo β-oxidation (fatty acid oxidation, FAO) to produce ATP, thereby meeting the energy demands of cancer cells [102,103,104]. Furthermore, lipid synthesis pathways, including the phospholipase-mediated hydrolysis of membrane lipids and de novo synthesis of essential FAs, are tightly regulated to support cancer cell survival and growth [105].

#### 2.2.2. Fatty Acid Uptake in Cancer Cells

Certain tumors exhibit the ability to uptake exogenous lipids from their surrounding microenvironment, highlighting the FA uptake pathway as a promising therapeutic target [106,107,108]. Key fatty acid transporters localized on the plasma membrane include cluster of differentiation 36 (CD36, also referred to as fatty acid translocase), the fatty acid transporter protein family (solute carrier family 27, SLC27), and fatty acid-binding proteins (FABPs) [106,109,110]. These transporters play critical roles in facilitating lipid uptake, and their dysregulation is frequently observed in cancer cells, contributing to tumor progression and metabolic adaptation [111,112,113]. Palmitic acid or fat-rich diets can specifically enhance the metastatic potential of cancer cells expressing high levels of CD36, suggesting the role of dietary lipid components and CD36 in the tumor metastasis process [114]. Fatty acid binding protein 4 (FABP4), an intracellular molecular chaperone for free FAs, is significantly upregulated during adipocyte differentiation. This protein plays a crucial regulatory role in multiple biological processes, including lipid metabolism, inflammatory responses, and angiogenesis, particularly in various types of solid tumors [115]. Under hypoxic conditions, tumor cells exhibit an increased uptake of FAs mediated by the expression of fatty acid binding proteins 3/7 (FABP 3/7) dependent on hypoxia-inducible factor-1α (HIF-1α), a process accompanied by a reduction in de novo fatty acid synthesis [116]. Under hypoxia, tumor cells upregulate extracellular fatty acid uptake through the enhanced expression of fatty acid transporters and oncogenic signaling activation [117,118,119]. Notably, hypoxia-induced proliferator-activated receptor γ (PPARγ) activation, mediated through direct transcriptional regulation by HIF-1, enhances both extracellular fatty acid uptake and triacylglycerol biosynthesis in tumor cells [120]. These adaptive responses collectively activate downstream signaling pathways that drive mitotic activity, thereby sustaining the uncontrolled proliferation and survival of tumor cells [121].

#### 2.2.3. Fatty Acid Synthesis in Cancer Cells

An effective strategy to reduce fatty acid concentration is to block their synthesis process. Citrate is a key node in FA metabolism, which needs to undergo a series of biochemical steps to convert carbon atoms from citrate into biologically active FAs [122]. These steps involve the participation of key enzymes, such as ATP citrate lyase (ACLY, ACL, or ATPCL), acetyl-CoA carboxylase (ACC), fatty acid synthase (FATCH or FAS), and acyl-CoA synthetase (also known as fatty acid CoA ligase, ACS, ACSL, or FACL) [97]. Inhibiting the activity of these key enzymes can effectively reduce the availability of FAs, thereby limiting the proliferation of cancer cells [123]. ACLY plays a critical role at the intersection of glucose metabolism and fatty acid metabolism by catalyzing the conversion of six-carbon citrate into oxaloacetate and two-carbon acetyl-CoA [124]. Consequently, the downregulation of ACLY weakens the cell’s ability to convert glucose metabolic products into lipids. In addition to participating in lipid metabolism in the cytosol, nuclear-localized ACLY can produce acetyl-CoA for histone acetylation and gene transcription regulation [125]. ACLY is transcriptionally upregulated by Sterol Regulatory Element-Binding Protein 1 (SREBP-1), inhibited by high concentrations of citrate through the homotropic allosteric regulation of ACLY [126,127]. ACC plays a crucial role in the fatty acid synthesis pathway, as it is the rate-limiting enzyme for FA synthesis, catalyzing the carboxylation of acetyl-CoA into malonyl-CoA [128]. ACC is one of the most stringently regulated enzymes in the fatty acid synthesis pathway [129]. ACC activity can be inhibited through phosphorylation by AMP-activated protein kinase (AMPK), and may also be modulated by a range of other kinases [130]. Malonyl-CoA decarboxylase (MCD) decarboxylates malonyl-CoA into acetyl-CoA, essentially reversing the reaction catalyzed by ACC [131]. FAS condenses one molecule of acetyl-CoA and seven molecules of malonyl-CoA into 16-carbon palmitate. Inhibition of FAS reduces fatty acid synthesis and induces malonyl-CoA accumulation, thereby inhibiting carnitine palmitoyltransferase 1 (CPT1)-mediated FAO, leading to cell cycle arrest and apoptosis in tumor cells [132,133,134]. The lipogenic gene Fatty Acid Synthase (FASN) is a significant therapeutic target, given its key role in mediating the synthesis of new FAs in most cancer cells, whereas most noncancer cells preferentially utilize exogenous FAs [135]. Free FAs following uptake are activated through CoA esterification catalyzed by members of the ACS family, yielding fatty acyl-CoA molecules that serve as substrates for either energy production via oxidation or the synthesis of various complex lipids [136]. Furthermore, bioactive FAs play a critical role in protein palmitoylation, a post-translational modification with significant biological implications in various cancers [137,138]. Stearoyl-CoA desaturase (SCD) catalyzes the introduction of a double bond at the C9 position of short-chain FAs (mainly converting stearoyl-CoA into oleoyl-CoA), and the inhibition of SCD function may lead to cell death in cancer cells by inducing the accumulation of unsaturated FAs [139,140]. In addition to directly targeting key enzymes, the FAS activities could be repressed by reducing transcription levels [141]. The main transcriptional regulator of FA synthesis is the SREBP-1 transcription factor. The mechanism by which SREBP-1 inhibition prevents cancer cell proliferation is through the reduction of SCD-1 expression and disruption of fatty acid desaturation, leading to lipotoxicity due to the abnormally high levels of saturated FAs [142,143,144].

#### 2.2.4. Fatty Acid Oxidation, Storage, and Utilization

The level of FAs in cancer cells can be reduced by increasing the rate at which they are degraded [145]. CPT1 serves as the rate-limiting enzyme in mitochondrial fatty acid β-oxidation, catalyzing the essential transport of fatty acyl-CoA into the mitochondrial matrix for subsequent oxidative degradation [106,146]. Inhibition of ACC 2 increases FA β-oxidation by decreasing malonyl-CoA levels, which is the direct product of ACC, and thereby alleviates the inhibition of CPT1 [147,148]. Therefore, weakening cancer cell proliferation by inhibiting ACC may also be partially due to the increase in FA degradation [149]. Peroxisome proliferator-activated receptor alpha (PPARα) is the main regulator of FAO, which promotes the breakdown of FAs by activating the expression of specific genes [150]. Studies have shown that PPARα activation inhibits tumor growth in several models [151].

Accumulating evidence suggests that the sequestration of FAs into neutral lipid storage pools, particularly triacylglycerols (TGs), may deplete the intracellular availability of fatty acid substrates required for membrane biogenesis and lipid-mediated signaling pathways, thereby potentially suppressing cellular proliferation [152]. In most cells, FAs are primarily stored in the cytosolic LDs as triglycerides, where LDs are organelles whose main function is lipid storage [153]. The main TG synthesis pathway is the glycerol phosphate pathway, which involves enzymes, such as glycerol-3-phosphate acyltransferase (GPAT), acylglycerol phosphate acyltransferase (AGPAT), phospholipid acid phosphatase (lipin or PAP), and diacylglycerol acyltransferase (DGAT), which catalyze the condensation reaction of FAs with glycerol-3-phosphate [154]. Except for the terminal enzyme DGAT, the products of all enzymes participate in phospholipid synthesis [97]. The DGAT enzyme catalyzes the reaction between diacylglycerol and FA-CoA, forming TG [155]. The DGAT enzyme plays a unique role in catalyzing the synthesis of triglycerides, being the only catalyst for the specific step [156]. Therefore, this enzyme becomes a key target for reducing free lipid levels by promoting lipid storage [97].

FAs produced by lipolysis can also serve as precursors for important signaling lipids [157]. Monoacylglycerol lipase (MAGL), a crucial regulator of lipid metabolism, has been established as a significant contributor to tumor progression through its dual role in facilitating FAO for energy production and promoting malignant transformation in cancer cells [158]. MAGL hydrolyzes the last FA in monoacylglycerol, leaving the glycerol backbone. In several invasive cancer cell lines and primary tumors, the expression and activity of MAGL are increased [159].

#### 2.2.5. Cholesterol Metabolism and Lipid Signaling in Cancer

Most cholesterol is stably located within the membrane, usually closely associated with sphingolipids and glycosylphosphatidylinositol-anchored proteins, forming dynamic nanoscale microdomains [160]. These microdomains can merge to form relatively ordered structures and play a key role in membrane trafficking, signal transduction, and the regulation of interactions between the host and pathogens [161,162]. Cholesterol is also a substrate for the synthesis of fat-soluble vitamins and steroid hormones [163]. In addition to its role in the structure and function of membranes, cholesterol gives rise to various oxysterols through enzymatic and nonenzymatic pathways, some of which are further metabolized into bile acids [164,165].

The cholesterol biosynthetic pathway is regulated by three critical components: SREBP-2, the primary transcriptional regulator of cholesterol biosynthesis, and two rate-limiting enzymes, 3-hydroxy-3-methylglutaryl-CoA reductase (HMGCR) and squalene monooxygenase [166,167]. Elevated cholesterol biosynthesis is a hallmark of numerous cancers [168]. In nutrient- and oxygen-deprived microenvironments, such as those found in glioblastomas, tumor cells exhibit a significant upregulation of SREBP-2 and its downstream target genes, including key enzymes in the mevalonate pathway, to meet the heightened demand for cholesterol and support tumor survival and growth [169]. Another transcription factor, RORγ, activates the cholesterol biosynthesis program and promotes the progression of triple-negative breast cancer [170]. Cholesterol biosynthesis also has a critical role in maintaining cancer stem cells by activating cellular signaling pathways downstream of sonic hedgehog, Notch, and receptor tyrosine kinases [171]. In addition, the upstream mevalonate pathway is oncogenic in a variety of cancers [172]. Compared with time- and energy-consuming de novo cholesterol synthesis, increasing cholesterol uptake might be more efficient for cancer cells. In terms of cholesterol uptake, dietary cholesterol is absorbed by the Niemann–Pick type C1–like 1 (NPC1L1) protein in the enterocyte cell membrane [102]. In cancer, the accumulation of cholesteryl esters (CEs) constitutes another common feature. CEs appear to act as a reservoir for cholesterol, allowing cancer cells to utilize these cholesterols when demand increases [173]. 

#### 2.2.6. Lipid Peroxidation, Ferroptosis, and Therapeutic Implications

Ferroptosis, a form of regulated cell death driven by lipid peroxidation (LPO), is characterized by the accumulation of lethal lipid peroxidation products (LPOs) [174]. These LPOs not only mediate cell death but also participate in cellular signaling pathways, serving as intermediates in the synthesis of eicosanoids, which regulate critical cellular processes, such as proliferation, survival, invasion, and migration [174,175]. During tumorigenesis and cancer progression, lipid metabolism plays a pivotal role in modulating ferroptosis. Cancer cells often display dysregulated apoptotic pathways and exhibit a heightened dependence on iron and lipid metabolism to support their proliferative demands [176,177]. This metabolic dependency renders cancer cells particularly vulnerable to iron-mediated cell death mechanisms, such as ferroptosis [178]. ROS, which are byproducts of aerobic metabolism, are continuously generated, metabolized, and eliminated in living organisms [179]. However, in cancer cells, dysregulated ROS production and LPO contribute to the initiation of ferroptosis, offering a potential therapeutic avenue for targeting tumor cells [180,181]. Studies have confirmed that acyl-coenzyme A synthetase 4 (ACSL4) exhibits overexpression in various types of cancer [182]. This protein plays a crucial role in regulating long-chain fatty acid metabolism and has been shown to potentiate cancer cell susceptibility to ferroptosis through facilitating the accumulation of LPO derivatives [183]. The p53 tumor suppressor gene is a key inhibitory factor in tumor development and is closely related to ferroptosis [184]. On one hand, p53 can increase the expression level of 15-LOX by promoting the expression of spermidine/spermine N1-acetyltransferase 1 (STA1) or by increasing the expression of GLS2 to promote the accumulation of LPOs, thereby enhancing ferroptosis. On the other hand, p53 can inhibit ferroptosis by reducing the accumulation of LPOs [185,186,187]. In summary, the interaction between ferroptosis and lipid metabolism contributes to p53-mediated tumor suppression [188]. Glutathione peroxidase 4 (GPX4) is a key regulator of ferroptosis, which inhibits the activity of cyclooxygenase (COX) and lipoxygenase (LOX) by reducing the level of LPO in cells. When GPX4 is inhibited, the conversion of GSH to glutathione disulfide (GSSH) will be attenuated, and LPOs will accumulate in tumor cells to trigger ferroptosis [174]. Moreover, the interaction between lipid metabolism and ferroptosis may affect the release of high mobility group protein 1 (HMGB1), thereby regulating tumor immunity. The phenotype and function of immune cells can be directly affected by ferroptosis [189].

### 2.3. Reprogramming of Amino Acid Metabolism in Cancer

Cancer cells have an increased requirement for amino acids to meet their rapid proliferation [190]. While the definition of essential amino acids (EAAs) and nonessential amino acids (NEAAs) is appropriate for normal cells, the classification does not apply to cancer cells [191]. Altered amino acids metabolism is common in tumors, and NEAAs usually become essential in tumors [192]. Targeting specific amino acid metabolic pathways represents a promising therapeutic strategy for controlling tumor progression and enhancing the efficacy of anticancer therapies [193]. Numerous studies reveal that various cancer cells exhibit a nutritional dependency on NEAAs [194]. Given that tumor cells can maintain rapid proliferation even under conditions of nutrient deficiency, their amino acid composition typically exhibits significant instability [195]. Extensive research has demonstrated that the 20 standard proteinogenic amino acids, including conditionally EAAs (such as glutamine, arginine), EAAs (e.g., branched-chain amino acids, tryptophan), and NEAAs (e.g., asparagine, aspartate), play a critical role in protein synthesis or energy metabolism activities within tumor tissues and act as metabolic products and regulators in promoting the proliferation of cancer cells [192].

Amino acid metabolism is one of the three major metabolic processes in the body, covering various pathways, such as glutamine (Gln) metabolism, serine metabolism, and glycine metabolism [196]. The main characteristics of tumor cells include metabolic reprogramming and immune evasion [197]. Amino acid metabolic reprogramming refers to the abnormalities in the rate of amino acid uptake, amino acid metabolic pathways, metabolites, or metabolism key enzymes in tumor cells during tumorigenesis [198]. An in-depth study of the abnormal metabolic processes of tumor amino acids is of significant importance for identifying future therapeutic targets for cancer [199].

#### 2.3.1. Glutamine

Gln is the most abundant type of amino acid in human plasma and also the one with the highest consumption by tumor cells [200]. Gln is not an EAA for normal cells; however, considering the significant increase in its demand by tumor cells, Gln becomes particularly crucial for tumor cells [201]. As an EAA required by tumor cells, Gln is involved in the rapid biosynthetic reactions of tumor cells [202]. Tumor cells exhibit a strong dependence on the utilization of Gln, a phenomenon known as Gln addiction [203]. Tumor cells acquire a large amount of Gln through both endogenous synthesis and exogenous uptake pathways. ASCT2 (SLC1A5), as the major Gln transporter in tumor cells, exhibits high expression in tumor tissues, and its expression level is significantly negatively correlated with patient prognosis [141]. Furthermore, tumor cells have the ability to synthesize Gln from glutamate and ammonia [204]. Gln synthetase (GS) is highly expressed in cancer cells to support their rapid proliferation [205].

After Gln is transported into the cell, it is converted into glutamate through the action of glutaminase (GLS), and then further into α-ketoglutarate (α-KG), which participates in the TCA [206]. The catabolic process of Gln begins with its conversion into glutamate under the catalytic action of GLS [207]. GLS1 and GLS2 as isozymes, play opposite roles in the process of tumor occurrence and development [208]. GLS1 exhibits oncogenic properties, whereas GLS2 is considered a tumor suppressor factor. Studies have found that in various types of tumors, including liver and colorectal cancer, the expression level of GLS1 is significantly increased, while the expression level of GLS2 is relatively low [209]. In the hypoxic microenvironment of tumors, HIF-1 activates the expression of GLS1 through its mechanism of action, thereby promoting tumor migration, invasion, and metastatic colonization [210]. In cancer cells, the expression of GLS and Gln metabolism is activated by the oncogenic transcription factor cMyc [211]. GSH and NADPH, which are produced by the Gln metabolism pathway, also participate in regulating the levels of intracellular ROS to maintain redox balance, thereby promoting the survival of tumor cells [212]. The accumulation of ROS leads to mitochondrial membrane hyperpolarization, thereby inducing ferroptosis. As a key metabolic precursor, Gln participates in the biosynthesis of uridine diphosphate-N-acetylglucosamine (UDP-GlcNAc) through the hexosamine biosynthesis pathway (HBP), playing crucial roles in mediating protein glycosylation processes and modulating endoplasmic reticulum stress responses in tumor cells [213]. Gln activates the mammalian target of rapamycin complex 1 (mTORC1) through the leucine transport mechanism, thereby promoting cell proliferation [214]. Glutamine-derived intermediates serve as essential precursors for the biosynthesis of purine and pyrimidine nucleotides, various NEAAs (including alanine, aspartate, serine, and proline), and reducing equivalents that drive OXPHOS [215]. 

#### 2.3.2. Serine and Glycine

Serine and glycine are NEAAs that are closely related in the biosynthetic process [216]. They serve as key precursors for the synthesis of structural units, including proteins, nucleic acids, and lipids that are crucial for cancer proliferation [217]. Functioning as crucial one-carbon donors in the folate-mediated metabolic pathway, serine and glycine, along with their associated enzymatic machinery, play pivotal roles in supporting tumor progression through facilitating nucleotide biosynthesis, epigenetic methylation processes, and the maintenance of cellular redox balance [218].

Serine, as a NEAA, plays a crucial role in the development of tumors through its synthesis process [219]. The initial step of the de novo serine synthesis pathway (SSP) is 3PG, which is produced during glycolysis and is catalyzed by phosphoglycerate dehydrogenase (PHGDH), phosphoserine aminotransferase (PSAT), and phosphoserine phosphatase (PSPH) [220]. PHGDH, the rate-limiting enzyme in the SSP, is frequently upregulated in various malignancies and metabolic disorders. This elevated expression enables tumor cells to overcome serine limitation by enhancing de novo serine biosynthesis, thereby providing essential one-carbon units to support nucleotide synthesis and sustain rapid tumor proliferation [221,222]. Serine palmitoyltransferase (SPT) is the rate-limiting enzyme in the synthesis of sphingolipids [223]. However, when serine synthesis is limited, SPT switches to using alanine as a substrate to synthesize cytotoxic deoxysphingolipids, thereby inhibiting the growth of tumor cells. Therefore, maintaining a certain level of serine is essential for tumor cells to evade cytotoxic inhibition [224]. Serine derived from 3-PG as well as exogenous serine can both be further converted into glycine under the action of serine hydroxymethyltransferase (SHMT) [225]. It is noteworthy that SHMT is a transcriptional target gene of c-Myc [226]. Glycine can also be formed from threonine through the action of threonine dehydrogenase and glycine C-acetyltransferase [227]. Subsequently, glycine is required for the synthesis of nucleic acids, proteins, and lipids, as well as for one-carbon metabolism, which is essential for DNA methylation [228]. Tumor cells primarily consume serine rather than glycine [229]. Cancer cells utilize a significant portion of glucose-derived carbon for the synthesis of serine, followed by the biosynthesis of folate and nucleotides. The loss of this metabolic bypass leads to cell death specifically in cancer cells [230]. SHMT (cytoplasmic, SHMT1; mitochondrial, SHMT2) catalyzes the transfer of a carbon unit from serine to tetrahydrofolate (THF), resulting in the formation of 5,10-methenyl-THF, which is crucial for nucleotide synthesis to promote rapid tumor proliferation [231].

#### 2.3.3. Arginine

Arginine (Arg) is a NEAA that plays a key role in the urea cycle [232]. This amino acid is involved in numerous important cellular metabolic pathways, including the biosynthesis of nitric oxide, nucleotides, proline, and glutamate [233]. The generation process of Arg involves citrulline, which is catalyzed by argininosuccinate synthetase (ASS1) and arginase (ASL) [234]. ASS1 is responsible for catalyzing the synthesis of argininosuccinate from L-citrulline and aspartate, and it is the rate-limiting enzyme in the de novo biosynthesis of Arg [235]. Subsequently, ASL converts argininosuccinate into L-Arg and fumarate, which links the metabolism of Arg to the energy metabolism of glucose production through the TCA cycle [236]. Unlike normal cells, most tumor cells lack the rate-limiting enzyme ASS for Arg synthesis, thus exhibiting a strong dependence on Arg [237].

Arg undergoes hydrolysis through the action of arginase, which is found in the cytoplasm as ARG1 and in the mitochondria as ARG2 [238]. In cancer cells, both isoforms of arginase are upregulated to guarantee the synthesis of polyamines [192]. Notably, ARG1 exhibits upregulation across a wider array of tumor types compared to ARG2. In the tumor environment, ornithine, which is upregulated, is further metabolized by ornithine decarboxylase (ODC) into polyamines, including putrescine, spermidine, and spermine [239]. These polyamines are a class of organic small molecule polycations that play a crucial role in DNA replication, translation, and cell proliferation processes [240]. Ornithine decarboxylase 1 (ODC1), as a key rate-limiting enzyme in the polyamine synthesis pathway, is highly activated in tumor cells and plays a significant role in tumor initiation and progression [241]. It is noteworthy that the products of Arg metabolism can participate in the urea cycle of tumor cells, and the urea cycle is regulated by the transcription factor p53 [242].

#### 2.3.4. Tryptophan

Tryptophan, an EAA for the human body, lacks a synthetic metabolic pathway within the human organism [243]. This amino acid is pivotal in the intrinsic malignant properties of tumors, capable of constraining the immune response to these neoplasms [244]. The primary metabolic pathway of tryptophan is the kynurenine pathway [245]. Free tryptophan, rather than tryptophan bound to albumin, can be catalyzed by indoleamine 2,3-dioxygenase 1 (IDO1) and tryptophan 2,3-dioxygenase (TDO) to produce kynurenine [246]. Within the kynurenine pathway, the production of a series of bioactive molecules affects the progression of tumors. Research indicates that the primary metabolite kynurenine can inhibit the proliferation of T cells and induce their apoptosis [247]. Under the catalytic action of kynurenine 3-monooxygenase (KMO) and kynureninase (KYNU), kynurenine can be further metabolized into NAD+ and alanine [192]. This kynurenine pathway is known as the de novo NAD+ synthesis pathway, which has significant resistance to oxidative stress and promotes the metastasis of cancer cells [248]. Beyond its metabolism through the kynurenine pathway, tryptophan is alternatively processed in enterochromaffin cells, where tryptophan hydroxylase 1 (TpH1) catalyzes its conversion into serotonin (5-hydroxytryptamine, 5-HT) through a distinct biosynthetic pathway [249]. 5-HT, also known as serotonin, has recently emerged as a growth factor for several human tumor cell types and the pattern of serotonin receptor subtype expression becomes dysregulated in several human tumors when compared with normal cells [250].

The increase in the kynurenine pathway is closely related to tumor progression [251]. In the kynurenine pathway, the high expression of IDO is associated with higher TNM stages and shorter overall survival [252]. The expression of IDO1 can be triggered as a counter-regulatory response to cytokines released by tumor-infiltrating immune cells (such as IL-1β and IL-6), and can also be maintained through intrinsic oncogenic signaling in the tumor [253]. TDO is an enzyme that catalyzes the same reaction as IDO1; its overexpression is also associated with poor prognosis [254].

#### 2.3.5. Asparagine and Aspartate

Aspartate, a fundamental substrate in the synthesis of nucleotides, fulfills various functions in the progression of tumors. As a metabolite that restricts tumor proliferation under hypoxic conditions, the concentration of aspartate correlates with markers of hypoxia [255]. Initially, aspartate is involved in the urea cycle within the cell [256]. Concurrently, the export of asparagine is accompanied by the reverse transport of serine, arginine, and histidine [257]. Asparagine also plays a role in regulating the epithelial-to-mesenchymal transition (EMT) to facilitate the colonization of tumor cells at distant metastatic sites [258].

The relative abundance of asparagine and Gln may exert a pivotal influence on tumor cells at the metastatic site [259]. Asparagine synthetase (ASNS) is the enzyme responsible for facilitating the conversion of aspartate into asparagine, while asparaginase (ASNase) catalyzes the hydrolysis of asparagine to produce aspartate. These two enzymes are crucial in regulating the balance of asparagine and aspartate within cells [260,261]. The limited cell membrane permeability of aspartate restricts its acquisition from the external environment, thereby hindering the synthesis of intracellular aspartate and the uptake of extracellular aspartate, thus suppressing tumor proliferation [262]. However, asparagine can be efficiently absorbed by tumor cells [263]. Tumor cells with elevated levels of ASNase can bypass the inherent limitations of aspartate by converting exogenously acquired asparagine into aspartate, thereby promoting tumor growth [264]. Some studies have utilized ASNase for systemic administration to reduce the availability of asparagine and prevent the rapid proliferation of cancer cells [265]. However, the majority of solid tumors produce asparagine by expressing ASNS, which diminishes their sensitivity to ASNase [266]. Additionally, aspartate can be synthesized through the catalytic action of glutamate oxaloacetate transaminase (GOT), utilizing the amino group from glutamate and oxaloacetic acid (OAA) derived from the TCA cycle [267]. Considering that cells are able to assimilate asparagine from the external environment or generate it via de novo synthesis and utilize it to produce aspartate, or generate aspartate through other pathways, it is imperative to target both these sources to effectively inhibit tumor cells.

#### 2.3.6. Proline

Proline (Pro) is the only imino acid with an α-amino group on a pyrrolidine ring, belonging to a special category of secondary amino acids in the proteinogenic group, and primarily involved in the synthesis of collagen [268]. As the most abundant protein in the body, collagen’s synthesis process is dependent on the metabolic pathway of Pro. In the metabolic pathway of Pro synthesis, glutamate is converted into δ-1-pyrroline-5-carboxylic acid (P5C) in the cellular mitochondria by δ-1-pyrroline-5-carboxylic acid synthase (P5CS) [269]. Ultimately, Pro is generated through the action of pyrroline-5-carboxylic acid reductase (PYCR), which catalyzes the conversion of P5C into proline [270]. Aldehyde dehydrogenase 18 family member A1 (ALDH18A1) is an ATP and NAD(P)H-dependent mitochondrial enzyme that promotes the synthesis of Pro and is upregulated in expression in various tumor cells [271]. Pro and Glu can be interconverted, with P5C and glutamate-γ-semialdehyde (GSA) serving as key intermediates in the conversion process [272].

In the process of promoting catabolism, Pro is converted into P5C by Pro dehydrogenase (PRODH) [273]. P5C spontaneously cyclizes to form GSA, which is then converted into Glu by pyrroline-5-carboxylic acid dehydrogenase (P5CDH) [274]. Additionally, GSA derived from Glu or Pro can be converted into ornithine, which is a precursor for Arg synthesis in the urea cycle [275]. PRODH, as a key rate-limiting enzyme in proline catabolism, is a p53-induced gene whose expression is downregulated in various tumors. However, other studies suggest that PRODH may also act as a tumor promoter, functioning as a double-edged sword in cancer [276]. 

#### 2.3.7. Branched-Chain Amino Acid

Branched-chain amino acids (BCAAs), encompassing isoleucine, leucine, and valine, are indispensable elements for cellular proliferation [277]. They serve as a nitrogen source for the creation of macromolecules, such as nucleotides and proteins, and yield metabolic byproducts, including glutamate, which are also integral to various metabolic processes [278]. BCAAs are pivotal in both normal and neoplastic cells and are categorized as EAAs. Since the human organism lacks the capability to produce BCAAs independently, the pertinent transport proteins are of particular significance. LAT1 (SLC7A5) and LAT2 (SLC7A8) are the principal transport proteins facilitating the uptake of BCAAs [192]. BCAAs influence protein synthesis by signaling cellular nutritional status or by serving as substrates for protein synthesis. The aggregation of BCAAs primarily stimulates the activation of mTORC1, thereby facilitating tumor development and growth. Variations in the concentrations of BCAAs in the peripheral blood may function as a potential biomarker for tumor onset [279].

The catabolism of BCAAs and their associated enzymes are intricately linked to the emergence and progression of neoplastic conditions. The breakdown of leucine, isoleucine, and valine is orchestrated by branched-chain amino acid transaminase (BCAT), resulting in the production of branched-chain α-keto acids (BCKAs), such as α-ketoisocaproate (α-KIC), α-keto-β-methylvalerate (α-KMV), and α-ketoisovalerate (α-KIV) [280]. As a pivotal enzyme in BCAA metabolism, BCAT upholds the relative equilibrium of BCAAs and BCKAs within cells [281]. α-KIC can be subsequently metabolized into acetyl-CoA, whereas α-KIV can be converted into succinyl-CoA. Regarding α-KMV, it can be further metabolized into both acetyl-CoA and succinyl-CoA. These metabolites are actively engaged in the TCA cycle [282]. Consequently, the catabolism of BCAAs is fundamental to the development of malignancies [283]. Furthermore, BCAAs are crucial for the synthesis of nucleotides, as they maintain the levels of ribonucleotide reductase regulatory subunit M2 (RRM2) [284].

### 2.4. Reprogramming of Nucleotide Metabolism in Cancer

Tumor cells exhibit various aggressive behaviors, including unrestricted proliferation, resistance to chemotherapy drugs, evasion of immune surveillance, and metastasis. These behaviors primarily depend on enhanced nucleotide metabolism. All proliferating cells require nucleotide building blocks (deoxyribonucleotide triphosphates, dNTPs) during the S-phase of the cell cycle to replicate their DNA, ensuring the accurate transmission of the complete genome to the next generation [285,286]. The uncontrolled growth signals provided by oncogenes induce replication stress, thereby promoting cancer progression. Numerous well-known carcinogenic drivers upregulate nucleotide biosynthesis, suggesting that this phenotype is essential for cancer initiation and progression [287].

In most organisms, purine nucleotide biosynthesis occurs through two distinct metabolic routes: (1) the de novo pathway, where nucleotides are synthesized through a multistep enzymatic cascade starting from 5′-phospho-α-D-ribose-1′-diphosphate (PRPP) as the initial substrate; and (2) the salvage pathway, which facilitates the metabolic recycling and interconversion of existing purine nucleotides, nucleosides, and free bases [288]. The initial stage of pyrimidine nucleotide de novo synthesis constructs the aromatic base (orotic acid), followed by the addition of the R5P group in a PRPP-dependent reaction. In contrast, purine nucleotide de novo synthesis starts with PRPP, and through a series of stepwise reactions, the aromatic base is built on the ribose scaffold [289]. Both de novo synthesis pathways rely on ATP; nitrogen and carbon that feed de novo nucleotide synthesis are provided by glutamine, aspartate, and several glucose-derived metabolites originating from the serine/glycine pathway, and one-carbon metabolism [290]. Pyrimidine nucleotide salvage is achieved through ATP-dependent steps catalyzed by uridine-cytidine kinase 1 (UCK1) and UCK2, deoxycytidine kinase (DCK), and thymidine kinase 1 (TK1) and TK2, resulting in the phosphorylation of (deoxy)nucleosides to form (d)NMPs. Purine deoxynucleotides are generated through the phosphorylation of deoxyadenosine and deoxyguanosine by deoxycytidine kinase (DCK), resulting in the production of dAMP and dGMP, respectively. This process is a key component of the purine nucleotide–nucleobase salvage pathway [287]. Conversely, the SAM domain and HD domain containing protein 1 (SAMHD1), which catalyzes the hydrolysis of dNTPs to deoxynucleosides, serves as a crucial regulator of intracellular dNTP pools in proliferating cells [291]. In various malignancies, including hematological cancers and solid tumors, SAMHD1 expression is frequently downregulated [292]. The dysregulation of functional enzyme expression has been closely associated with carcinogenesis, given the well-established understanding that imbalanced dNTP pools can induce genomic instability and disrupt cell-cycle progression, ultimately promoting cancer cell proliferation [293].

The abnormal activation of the PI3K/AKT/mechanistic target of the mTORC1 signaling pathway is a common feature in various tumors, leading to a reprogramming of cellular metabolism, which promotes biosynthesis and biomass generation [294]. The mechanistic target of mTORC1 is activated downstream of the PI3K/AKT pathway and promotes the S6 kinase 1 (S6K1)-dependent phosphorylation of carbamoyl-phosphate synthetase 2, aspartate transcarbamoylase, and dihydroorotase, thus enhancing de novo pyrimidine synthesis [295,296]. Additionally, mTORC1 activation stimulates the de novo synthesis of purines and pyrimidines by increasing cellular bicarbonate abundance [297]. The tumor suppressor PTEN negatively regulates the PI3K-Akt pathway, and PTEN mutations or deletions activate AKT and mTORC1 signaling, promoting cell growth [298]. In PTEN-deficient cells, de novo pyrimidine synthesis is enhanced through mTORC1-dependent CAD phosphorylation [295]. Compared to the regulation of de novo pyrimidine synthesis, mTORC1 signaling promotes purine nucleotide de novo synthesis via long-term mechanisms by inducing the transcription factors ATF4 and MYC, both of which regulate the expression of specific metabolic enzymes involved in purine nucleotide de novo synthesis or required for it. For example, during mTORC1 activation, ATF4 enhances the production of enzymes involved in the serine/glycine synthesis pathway and mitochondrial tetrahydrofolate cycle, both of which produce glycine and one-carbon units essential for purine nucleotide de novo synthesis [299]. Extracellular signal-regulated kinase (ERK), a member of the mitogen-activated protein kinase (MAPK) family, plays a crucial role in signal transduction cascades by efficiently transmitting extracellular signals to intracellular targets [300]. Consequently, the MAPK cascade constitutes a central signaling mechanism that regulates fundamental cellular processes, including cell proliferation, differentiation, and response to environmental stresses [301]. MYC, as a downstream transcription factor of the RAS-ERK signaling pathway, regulates the expression of genes involved in purine and pyrimidine de novo synthesis. ERK activation enhances MYC’s transcriptional activity, and since MYC directly regulates the expression of genes involved in pyrimidine and purine synthesis, it is considered a key regulator of nucleotide metabolism [302].

Dihydroorotate dehydrogenase (DHODH) is a critical enzyme in the de novo synthesis of uridine monophosphate (UMP), which is essential for pyrimidine nucleotide biosynthesis [303]. DHODH may also play an important role in cancer cell migration during metastasis. Since all pyrimidine nucleoside triphosphates (dNTPs) are derived from UMP, DHODH inhibition leads to the depletion of these nucleotides [304]. In the purine de novo synthesis pathway, inosine monophosphate (IMP), an intermediate, can be converted into adenosine monophosphate (AMP) or guanosine monophosphate (GMP). The GMP production arms of this pathway are targeted by several approved drugs [305]. As the most abundant RNA component in cells, ribosomal RNA (rRNA) undergoes substantial quantitative and qualitative alterations in cancer cells, manifested by enhanced rRNA production and pronounced nucleolar hypertrophy, thereby facilitating accelerated protein synthesis [306,307]. While dNTPs are essential for DNA synthesis, NTPs are required for RNA transcription, and inhibitors blocking the formation of both dNTPs and NTPs (such as DHODH or IMPDH inhibitors) are expected to limit rRNA transcription and translation. Furthermore, studies have shown that DHODH inhibition significantly reduces the growth of PTEN-deficient cells [308]. ASS1, a key enzyme in the urea cycle, catalyzes the synthesis of argininosuccinate from citrulline and aspartate [231]. In cancer cells with downregulated ASS1 expression, the depletion of aspartate is reduced, leading to the accumulation of intracellular aspartate pools [309]. These accumulated aspartate pools are subsequently utilized by CAD enzymes to produce N-carbamoylaspartate, thus promoting pyrimidine synthesis to support cancer cell proliferation [310]. However, in tumor cells with impaired urea cycle function, excessive pyrimidine synthesis may lead to genomic instability and mutation, potentially altering the tumor’s sensitivity to immune checkpoint inhibitors [311]. SAMHD1 catalyzes the α-phosphohydrolysis of dNTP molecules, generating the corresponding deoxynucleosides and inorganic triphosphates [312]. Notably, the triphosphate group in dNTP molecules is indispensable for DNA polymerase-mediated DNA synthesis, and SAMHD1’s catalytic activity is regulated by nucleotide abundance [313,314]. Nucleotide-metabolizing enzymes, such as ribonucleotide reductase (RNR), which plays a role opposite to SAMHD1 in nucleotide metabolism, reduce NDPs to dNDPs. RNR exhibits a broad substrate specificity, and similarly, SAMHD1’s catalytic site can accommodate all typical dNTPs, including dGTP, dATP, dCTP, dTTP, and dUTP, and subsequently hydrolyze them [312]. This phenomenon aligns with the view of SAMHD1 as a major regulator of the dNTP pool in human cells [315]. Methylenetetrahydrofolate dehydrogenase 2 (MTHFD2), a mitochondrial bifunctional enzyme with both dehydrogenase and cyclohydrolase activities, plays a pivotal role in one-carbon metabolism [316]. This enzyme facilitates the conversion of serine-derived one-carbon units into methylene-THF (CH2-THF) within the mitochondrial compartment. The CH2-THF complex subsequently undergoes oxidation to generate formate, which is then exported to the cytosol. In the cytosolic compartment, this formate serves as a critical one-carbon donor for essential biosynthetic processes, including de novo purine biosynthesis, thymidylate production, and methionine synthesis [317]. MTHFD2 is one of the most upregulated metabolic enzymes in cancer, making it an attractive anticancer target [318].

## 3. Nanoparticle Drug Delivery: Properties, Enrichment, and TME-Responsive Release in the Tumor Microenvironment

**Figure 2:**
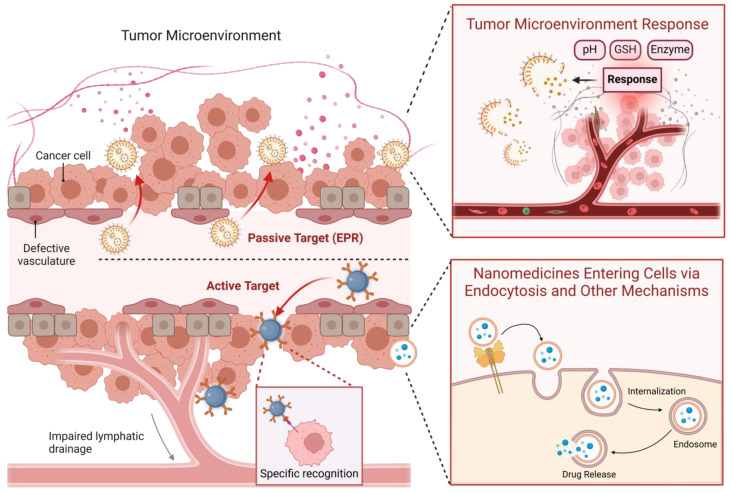
The targeting mechanisms of nanodrugs in the tumor microenvironment (TME). Nanoparticle-based drugs primarily achieve passive targeting of the TME through the enhanced permeability and retention (EPR) effect, leading to accumulation within the TME. Additionally, specific modifications on the nanoparticle membrane enable active targeting through tumor cell-specific recognition. Once entering the TME, some nanoparticles are designed to respond to the physicochemical characteristics of the microenvironment, enabling controlled drug release and increasing the concentration of free drugs in the microenvironment. Other nanoparticles, through processes, such as endocytosis, enter tumor cells and, after lysosomal escape, exert their therapeutic effects. Created with Biorender.com (accessed on 24 February 2025).

Compared to traditional drugs, nanoparticle-based drugs typically exhibit higher drug activity, greater potency, and lower toxicity. Additionally, the TME presents unique physiological characteristics, such as increased vascular permeability for macromolecules, poor lymphatic drainage, and specific molecular markers, which provide opportunities for targeted drug delivery using nanomedicines [319]. The administration of nanoparticle-based therapeutics involves two key processes: the first is the targeting of the TME by nanoparticles within the systemic circulation, and the second is the internalization of the drug by tumor cells within the microenvironment. By leveraging the unique properties of nanoparticles to achieve targeted delivery to the TME, drug accumulation and retention within the tumor can be improved, thereby maximizing therapeutic efficacy and minimizing toxicity to normal tissues. In addition, regarding the release of nanoparticle drugs, the microenvironment-targeted release approach utilizes the characteristics of the TME to achieve targeted drug release and maximize therapeutic efficacy (Figure 2) [320].

### 3.1. Physicochemical Characteristics of Nanoparticle-Based Therapeutics

Many traditional drugs suffer from poor water solubility, rapid metabolism, and low bioavailability, which prevent them from meeting clinical needs. However, by encapsulating these drugs in nanoparticles to create the corresponding nanodrugs, their solubility can be improved, thereby increasing drug utilization and reducing the dosage required, thus lowering drug toxicity [321]. For example, curcumin, a polyphenolic compound extracted from turmeric, has anti-inflammatory and anticancer properties. When encapsulated in liposomes or polymer nanoparticles, its solubility is improved, and its biological toxicity is reduced, showing enhanced therapeutic effects [322]. Another example is Amphotericin B, a polyene antibiotic widely used for systemic fungal infections. However, Amphotericin B carries a risk of nephrotoxicity. Studies have shown that when Amphotericin B is encapsulated in a lipid-based carrier system, its toxicity is reduced, and it becomes better suited for clinical treatment [323].

### 3.2. Enrichment of Nanodrugs in the Tumor Microenvironment

The main advantage of nanodrugs is their ability to accumulate and retain within the TME, enabling targeted delivery to the tumor site, increasing local drug concentration, and reducing toxicity to normal tissues [324]. Currently, there are two main strategies for targeting the TME with nanodrugs: one is passive targeting based on the inherent properties of the nanodrugs, and the other involves the surface modification of the nanodrugs to enable active binding to tumor cells, thereby achieving active targeting.

#### 3.2.1. Passive Targeting

Passive targeting refers to the accumulation of nanomedicines at tumor sites due to the unique physiological characteristics of tumor tissue, such as the enhanced permeability and retention (EPR) effect. This approach does not rely on specific ligand–receptor interactions but instead depends on the physicochemical properties of the nanomedicines (such as particle size, surface charge, circulation time, etc.) and the characteristics of the TME to achieve targeted accumulation in the tumor site [325]. In the unique structure of solid tumors, such as the high vascular system, vascular structural defects, and impaired lymphatic drainage, the EPR effect is significantly enhanced. In addition, there is considerable heterogeneity in the EPR effect across different tumor types and individual differences [326]. Due to the distinctive nature of the EPR effect, not all nanomedicines exhibit this phenomenon [327]. The EPR effect is more pronounced in experimental animal tumor models than in human tumors, meaning that compared to animal tumors, the efficiency of nanoparticle delivery in human tissues is low. One important reason for this phenomenon is that most nanoparticle drug products are developed using small rodent tumor models, where the tumors are either induced by carcinogens or genetically engineered, which differs from tumor formation in humans. Therefore, nanodrugs developed in these small rodent tumor models have limitations. The primary approach to overcoming this issue in the future is to develop new drug testing models, thus compensating for the inability of nanoparticle drugs to perform perfectly in clinical settings [328].

Doxil and Caelyx are two nanoparticle drugs that can accumulate in preclinical and human tumors, moderately improving the delivery efficiency of nanoparticles in human tissues [329]. Doxil is the first FDA-approved nanomedicine, which achieves passive targeting through the EPR effect. Its PEGylated liposomes extend plasma circulation time (approximately 90 h), selectively accumulate in tumor tissues, and release doxorubicin slowly, enhancing antitumor efficacy while reducing toxicity and side effects [330]. Caelyx (Stealth liposome-encapsulated doxorubicin) achieves passive targeting through the EPR effect. Its Stealth^®^ liposomes (approximately 100 nm) allow prolonged circulation in the bloodstream, reducing drug exposure to normal tissues. Meanwhile, they penetrate tumor tissues through vascular gaps, lodge in the interstitial spaces among tumor cells, and release the drug slowly. Studies have demonstrated that compared to conventional doxorubicin, Caelyx achieves significantly higher drug concentrations in tumor tissues, effectively enhancing antitumor activity. Moreover, the controlled drug release of Caelyx reduces peak plasma drug concentrations, significantly minimizing cardiac toxicity and improving the safety and tolerability of the treatment [331].

#### 3.2.2. Active Targeting

Active targeting involves functionalizing the surface of nanoparticles with specific ligands that enable them to bind to receptors on target tissues or cells, thereby achieving selective drug delivery and enhanced therapeutic effects [332]. After modification, nanodrugs may enter cells through mechanisms, such as phagocytosis, pinocytosis, and receptor-mediated endocytosis [333]. This mechanism relies on the recognition and binding of targeting carriers to specific molecular markers in the target area. It may also involve responsive materials to ensure precise drug release at the target site. This technique significantly enhances drug concentration at the target, improving therapeutic efficacy while reducing nonspecific damage to normal tissues, making it particularly promising in tumor therapy and other fields [334]. For example, the density of tumor-associated macrophages (TAMs) has been shown to have a significant linear correlation with the accumulation of nanomedicines in tumors, and the active targeting of TAMs can enhance the accumulation of nanomedicines in tumors. In the A431, MLS, and CT26 tumor models, the fraction of TAMs increased from 2.2% to 5.1% and 7.7%, respectively, with a corresponding significant increase in nanomedicine accumulation. By actively targeting TAMs, not only can they serve as a reservoir for nanomedicines, but enhancing TAM density also optimizes the accumulation efficiency of nanomedicines in tumors [335].

BIND-014 is a PSMA (prostate-specific membrane antigen)-targeted nanomedicine that achieves specific binding to tumor cells or tumor-associated vasculature by modifying its nanoparticle surface with small-molecule PSMA-targeting ligands, thereby enhancing the accumulation of docetaxel at tumor sites. Its polylactic acid core allows for the slow release of the drug, while the PEG-modified outer layer reduces immune clearance and prolongs circulation time in the bloodstream. Clinical studies have shown that BIND-014 significantly increases therapeutic efficacy in tumor tissues while reducing side effects, demonstrating superior pharmacokinetic properties and antitumor activity [322]. Trastuzumab-conjugated nanoparticles are designed for the active targeting of HER2-positive tumor cells. They covalently link thiolated trastuzumab to HSA nanoparticle surfaces using a PEG-based cross-linker. This enables the nanoparticles to specifically bind to HER2 receptors on tumor cells, enhancing drug accumulation at tumor sites. The experiments demonstrate that these nanoparticles have a significantly higher uptake in HER2-positive cells compared to nontargeted nanoparticles, while showing no such difference in HER2-negative cells. This indicates their ability to actively and specifically target HER2-positive tumor cells, holding great promise for precise cancer treatment with reduced side effects [336].

### 3.3. Drug Release in Response to Tumor Microenvironment

Nanomedicine delivery systems can rely on the unique characteristics of the TME to achieve controlled drug release. The TME is distinguished by specific features, including low pH, elevated GSH levels, hypoxia, and the overexpression of certain enzymes, all of which can be exploited for targeted drug delivery. By leveraging these factors, nanomedicines can deliver drugs specifically to the tumor site, enhancing therapeutic efficacy while minimizing systemic toxicity to healthy tissues.

#### 3.3.1. pH-Responsive Systems

The acidic nature of the TME, primarily resulting from the high metabolic activity of tumor cells and the accumulation of lactic acid, serves as an effective trigger for pH-responsive drug release. Nanocarriers equipped with pH-sensitive materials undergo structural changes in acidic conditions, leading to the controlled release of the encapsulated drug. This approach increases the drug concentration at the tumor site and reduces the systemic toxicity to surrounding healthy tissues [337,338]. For example, Dox is encapsulated in nanoparticles made of poly (d, 1-lactic-co-glycolic acid) (PLGA) and combined with mesenchymal stem cells, utilizing the pH sensitivity of the system for controlled drug release. Studies have shown that these PLGA-Dox nanoparticles exhibit significant release differences under various pH conditions: the drug release rate is significantly higher in acidic environments and remains low in neutral pH. This pH-responsive property helps achieve efficient drug delivery within tumor tissues, enhancing antitumor effects [339]. In addition, glycyl-phenylalanyl leucyl glycine (GFLG) is linked to the backbone of a copolymer of hydroxypropyl methacrylate (HPMA) and oligo (ethylene glycol) methacrylate (OEGMA), and then combined with certain chemicals to obtain DOX/nifuroxazide (NFX) co-loaded micelles (CLMs). When the micelles encounter the acidic TME, the hydrazone and GFLG bonds are cleaved, leading to the pH-responsive release of DOX and NFX, thereby enhancing the therapeutic effect [340].

#### 3.3.2. GSH-Responsive Systems

GSH is an abundant intracellular antioxidant, found at significantly higher concentrations in tumor cells compared to normal tissues, making it an ideal trigger for controlled drug release in cancer therapy. Nanoparticles sensitive to GSH have been designed to exploit this disparity, typically incorporating disulfide bonds (S–S) or other GSH-sensitive linkers that are cleaved in the high-GSH tumor environment, leading to the release of the encapsulated therapeutic agents. One notable example is GSH-responsive poly-resveratrol (PRES) nanoparticles, which are capable of overcoming multidrug resistance by efficiently releasing drugs, like paclitaxel, in response to the elevated GSH levels in tumor cells. These nanoparticles exhibit enhanced stability in the bloodstream but rapidly degrade in the tumor environment, facilitating targeted drug delivery and improving therapeutic efficacy. This strategy ensures minimal systemic toxicity while maximizing the therapeutic impact at the tumor site [341].

#### 3.3.3. Enzyme-Responsive Systems

Enzyme-responsive drug delivery systems represent another exciting approach to targeted cancer therapy. TME often exhibit overexpressed enzymes, such as phospholipases and proteases, which are involved in various pathological processes, including tumor progression and metastasis. These enzymes can be exploited to trigger the release of drugs from nanocarriers by cleaving enzyme-sensitive linkers that are incorporated into the nanocarriers [342,343]. There are various enzyme-responsive delivery systems. For example, one study combined a glucuronic acid trigger, a self-immolative linker, the potent monomethyl auristatin E (MMAE), and a maleimide-functionalized side chain into a single entity. This drug delivery system binds to serum albumin, and the conjugated macromolecule accumulates in the TME. The β-glucuronidase in the microenvironment cleaves the glucuronic acid moiety, triggering the release of MMAE, thereby achieving enzyme-responsive drug release [344].

## 4. Nanomedicine Targeting Tumor Metabolism

**Figure 3:**
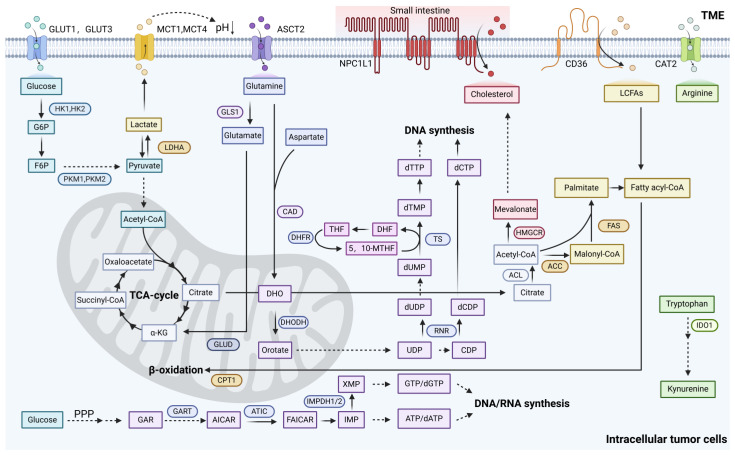
The key metabolic pathway components and targets for nanomedicine-based targeting (rectangles represent components, rounded rectangles represent enzymes, and dashed lines and multiple arrows indicate omitted multistep reactions). The primary mechanisms of action of nanomedicines currently include blocking the intake of substances, depleting critical components, and targeting rate-limiting or key catalytic enzymes within metabolic pathways. GLUT1 (Glucose Transporter 1), GLUT3 (Glucose Transporter 3), MCT1 (Monocarboxylate Transporter 1), MCT4 (Monocarboxylate Transporter 4), ASCT2 (Ascitic Amino Acid Transporter 2), NPC1L1 (Niemann-Pick C1-Like 1), HK1 (Hexokinase 1), HK2 (Hexokinase 2), G6P (Glucose-6-Phosphate), F6P (Fructose-6-Phosphate), PKM1 (Pyruvate Kinase M1), PKM2 (Pyruvate Kinase M2), LDHA (Lactate Dehydrogenase A),α-KG (Alpha-Ketoglutarate), GLS1 (Glutaminase 1), CAD (Carbamoyl Phosphate Synthetase 2), DHO (Dihydroorotate), DHODH (Dihydroorotate Dehydrogenase), GLUD (Glutamate Dehydrogenase), RNR (Ribonucleotide Reductase), TS (Thymidylate Synthase), DHF (Dihydrofolate), THF (Tetrahydrofolate), DHFR (Dihydrofolate Reductase), 5,10-MTHF (5,10-Methylenetetrahydrofolate), GAR (Glycinamide Ribonucleotide), GART (Glycinamide Ribonucleotide Transformylase), AICAR (Aminoimidazole Carboxamide Ribotide), ATIC (Aminoimidazole-4-carboxamide ribonucleotide transformylase/IMP cyclohydrolase), FAICAR (Formyl-Aminoimidazole Carboxamide Ribotide), IMP (Inosine Monophosphate), IMPDH1/2 (Inosine Monophosphate Dehydrogenase 1/2), XMP (Xanthosine Monophosphate), CD36 (Cluster of Differentiation 36), LCFAs (Long-Chain Fatty Acids), CAT2 (Cationic Amino Acid Transporter 2), HMGCR (3-Hydroxy-3-Methylglutaryl-CoA Reductase), ACL (ATP-Citrate Lyase), ACC (Acetyl-CoA Carboxylase), FAS (Fatty Acid Synthase), IDO1 (Indoleamine 2,3-Dioxygenase 1), CPT1 (carnitine palmitoyltransferase 1). Created with Biorender.com (accessed on 24 February 2025).

Due to the high proliferation and replication demands of tumor cells, they require aberrant metabolism to meet their material and energy needs. Therefore, selectively targeting tumor metabolism has become an ideal strategy for cancer treatment [345]. Current approaches commonly involve targeting the tumor metabolism of glucose, lipids, amino acids, and nucleotides. In order to achieve more efficient drug delivery to the TME, an increasing number of nanomedicines are being developed for precise drug delivery and the disruption of tumor metabolism [346]. The development of nanoparticle-based drugs may follow three directions: reducing the intake of metabolic precursors, consuming substances already produced in the microenvironment, and targeting the inhibition of key enzymes involved in metabolism (Figure 3).

### 4.1. Nanoparticle-Based Drugs Targeting Tumor Glucose Metabolism

Glucose is a crucial substrate for energy production and carbohydrate metabolism in the body. Tumor cells require a significant amount of glucose to generate the necessary metabolic precursors and energy for their survival. Due to the Warburg effect, tumor cells preferentially rely on aerobic glycolysis for energy production. Therefore, using nanoparticle-based drugs to limit glucose uptake, reduce glucose levels in the TME, and disrupt glycolytic signaling pathways can effectively impair energy supply, exerting an antitumor effect.

#### 4.1.1. Nanomedicines Inhibit GLUTs to Reduce Glucose Uptake in Tumor Cells

To meet their metabolic demands, tumor cells require the uptake of large amounts of glucose. Through the process of glycolysis, glucose is catalyzed to generate various carbon intermediates, which serve as the foundational materials for the synthesis of various macromolecules [347]. Research has shown that GLUTs are highly expressed in tumor cells, leading to increased glucose uptake, and glucose deprivation could serve as a potential therapeutic approach. Nanomedicines can be used to target and inhibit GLUTs, thereby reducing glucose uptake in tumor cells, ultimately disrupting energy production and biosynthesis within the tumor cells. Ahmad Abolhasani et al. developed glucose-modified PLGA nanoparticles and chitosan nanoparticles (GPNPs and GCNPs), which act as blockers by specifically binding to the overexpressed glucose transporters (Gluts) in tumor cells, thereby preventing the facilitated transport of glucose. The results demonstrated that the drug significantly increased apoptosis rates in human colon cancer cells, effectively blocking nutrient entry into the cells and subsequently inhibiting tumor cell growth [348]. Songyi Lee et al. developed an amphiphilic conjugate based on hyaluronic acid-ceramide-dopamine (HACE-d) and prepared HACE-d-based nanoparticles containing phloretin, a glucose transporter GLUT1 inhibitor. The interaction between HA and the CD44 receptor enabled active targeting, while the mussel-inspired properties of dopamine enhanced cell adhesion, improving nanoparticle penetration in tumor tissue and cellular uptake. This facilitated the efficient delivery of phloretin, which inhibited GLUT1, and reduced glucose uptake in tumor cells, leading to inhibited tumor cell growth [349]. Congfei Xu et al. used cationic lipid-assisted poly (ethylene glycol)-b-poly(D,L-lactic acid) nanoparticles (PEG-PLA) as a carrier to efficiently deliver siRNA into U87MG and U251 glioma stem cells and numerous glioma cells. The nanoparticles carrying specific siRNA targeting GLUT3 (NPsiGLUT3) significantly reduced GLUT3 expression in glioma stem cells. Knockdown of GLUT3 resulted in the inhibition of glucose uptake and metabolism in tumor cells, thereby reducing lactate production and increasing ROS generation, which significantly suppressed tumor cells and tumor stem cells, inducing cell apoptosis [350].

#### 4.1.2. Nanomedicines for Glucose Depletion

The upregulation of aerobic glycolysis in tumor cells makes them more sensitive to changes in glucose and ATP levels. Therefore, the strategy of depleting high-energy substances within tumors to exhaust tumor energy, known as “starvation therapy”, can be used to mediate tumor cell death. Glucose oxidase (GOx) specifically catalyzes the conversion of glucose to gluconic acid and H_2_O_2_ in the presence of O_2_, thereby disrupting the redox balance of tumor cells. This leads to tumor cell starvation by depleting glucose and O_2_, resulting in the disruption of glucose metabolism pathways [351]. Xirui Duan et al. co-assembled GOx with indocyanine green (IR820) and other agents, utilizing the photothermal properties of IR820 to provide the appropriate temperature and oxygen supply for the enzymatic reaction of GOx, thereby promoting intracellular glucose consumption. This photothermal and starvation synergistic therapy significantly reduced the recurrence and metastasis of colorectal cancer [352].

#### 4.1.3. Nanomedicines for Inhibiting the Glycolytic Pathway

There are three key rate-limiting enzymes in glycolysis: HK, PFK-1, and Pyruvate Kinase. The current research on nanomedicines primarily focuses on targeting HK and Pyruvate Kinase. 3-Bromopyruvate (3-BrPA) is a multitarget aerobic glycolysis inhibitor that can inhibit HK 2 and GAPDH, further suppressing the production of lactate and ATP. Xuan Meng et al. employed ZIF-8 as a carrier for loading GOx and 3-BrPA, leveraging the pH- and ATP-responsive properties of ZIF-8 to selectively target the TME. 3-BrPA enhances oxygen concentration by inhibiting glycolysis and the TCA, leading to pyruvate depletion and augmenting the effect of GOx. The combination of GOx and 3-BrPA synergistically inhibits glycolysis, resulting in decreased ATP, lactate, and GSH, further impairing mitochondrial function, while disrupting tumor cell energy metabolism and redox balance, thereby achieving tumor cell killing [353]. Manling Chen et al. prepared metformin as a nanoparticle derivative (MA-dots), which retained the anticancer properties of metformin while also providing a targeted delivery platform for 2-deoxy-D-glucose (2-DG, a glycolysis inhibitor). In the acidic TME, the MA-dots complex decomposes to release 2-DG, which inhibits the key enzyme HK2 in glycolysis, while metformin inhibits mitochondrial OXPHOS. This dual mechanism significantly reduces ATP production in tumor cells, thereby promoting tumor cell apoptosis [354].

Pyruvate kinase catalyzes the conversion of phosphoenolpyruvate to pyruvate, providing precursor molecules for key metabolic pathways, such as lactate metabolism. PKM2 is abundantly expressed and upregulated in tumor tissues, which renders it a promising target for therapeutic interventions [355]. Rui Yang and colleagues synthesized a coral-shaped DNA-FeS2-DA nanoparticle, using double-stranded DNA rich in ‘GAG’ and ‘GC’ sequences as a template and poly-dopamine as an adhesive. Curcumin (CUR) was successfully loaded into the DNA-FeS2-DA, forming the CUR@DNA-FeS2-DA nanocomposite. This nanocurcumin can efficiently enter the mitochondria of cancer cells and gradually release curcumin in a weakly acidic environment. The CUR@DNA-FeS2-DA nanocomposite effectively inhibits the expression of PKM2 and FAS in MCF-7 cells, thereby disrupting the energy metabolism of cancer cells. Due to its desirable photothermal effect, stability, and targeting properties, it holds great potential as a novel anticancer therapeutic agent [356]. Juanjuan Dang et al. designed a guanidine-rich, spherical helical polypeptide (DPP) with multivalency-assisted strong membrane-penetrating ability, enabling the efficient delivery of targeted PKM2 siRNA into tumor cells to specifically inhibit tumor glycolytic metabolism. DPP was capable of loading infrared-absorbing agent (ICG) into its internal cavity and forming positively charged nanocomplexes by condensing siRNA targeting PKM2. These nanocomplexes were further coated with human serum albumin to enhance their stability in circulation, prolong blood circulation time, and improve tumor targeting. The effective silencing of PKM2 was achieved both in vitro and in vivo, resulting in the inhibition of tumor glycolysis and the subsequent downregulation of HSP expression, overcoming the thermoresistance of tumor cells and enhancing ICG-mediated photothermal ablation [357].

### 4.2. Nanoparticle-Based Approach for Disrupting Lactate Metabolism

Tumor cells have distinct energy requirements compared to most normal cells, primarily relying on aerobic glycolysis to convert glucose into lactate. While lactate is often considered a metabolic byproduct in the TME, studies have shown that when tumor cells shift their metabolism towards OXPHOS, lactate can serve as an alternative energy source through the TCA cycle, closely associated with tumor progression and immune suppression [358]. Lactate reprogramming of the TME has profound effects on the phenotypes of cancer cells, making the inhibition of lactate metabolism a promising strategy for cancer therapy [359].

#### 4.2.1. Nanodrugs Inhibiting Key Enzymes of Lactate Production

The primary lactate produced in mammals is L-lactate. LDHA uses NADH as a reductant to convert pyruvate to L-lactate, making it a key catalytic enzyme in lactate metabolism. Inhibiting LDHA reduces lactate production and aids tumor treatment [360]. Yuxue Zhang et al. used polymer-based cationic lipid-assisted nanoparticles (CLANs) for siRNA delivery, downregulating LDHA expression, and reducing lactate secretion, which reversed immune suppression and significantly improved antitumor effects when combined with PD-1 checkpoint blockade [361]. Lijun Hu et al. used the cationic polymer APEG-PAsp (PEI) (PAPEI) for siRNA delivery to silence LDHA, inhibiting the M2-like polarization of tumor-associated macrophages and weakening immune suppression, effectively enhancing the anticancer effects of oxaliplatin (OXA) in colorectal cancer [362].

#### 4.2.2. Nanodrugs for Reducing Lactate Efflux

Inhibiting lactate efflux-related receptors reduces subsequent lactate transport and helps reverse the high lactate concentration in the TME. Lirong Tian et al. used liposomes to deliver lonidamine and syrosingopine, constructing L@S/L. Lonidamine reduces lactate production by affecting glycolytic metabolic pathways, while syrosingopine inhibits the key protein expression of lactate transporters MCT-4, thereby reducing lactate efflux. This dual action reversed immune suppression in the TME and further inhibited tumor growth [363]. Zhaoxia Chen et al. employed mesoporous silica nanoparticles (MSNs) as the carrier material, coating them with MnO2 and delivering metformin and fluvastatin sodium. Metformin regulates the pyruvate metabolic pathway, promoting lactate production, while fluvastatin sodium inhibits MCT4, reducing lactate efflux and increasing intracellular lactate accumulation, leading to an acidic intracellular environment that kills tumor cells. As lactate efflux is restricted, the lactate concentration in the TME decreases, and the migration and resistance abilities of tumor cells are significantly weakened [364].

#### 4.2.3. Nanodrugs for Lactate Consumption

While inhibiting lactate dehydrogenase or reducing lactate efflux can suppress lactate production, it does not reduce the lactate already produced in the TME. Lactate oxidase can consume lactate to produce pyruvate and hydrogen peroxide, depleting lactate and generating toxicity against tumors, showing great potential [365]. Zhan Zhang et al. used lactate oxidase (LOX) and mitochondrial respiration inhibitor atovaquone (ATO) to create lactate depletion and inhibit mitochondrial function, alleviating tumor hypoxia and acidic TME while reversing tumor immune suppression [366]. Shengming Wu et al. used hollow Fe_3_O_4_ catalytic nanocarriers to deliver lactate oxidase (LOD) and syrosingopine. By inhibiting MCT-1 and MCT-4, they suppressed lactate efflux and consumed lactate in the TME, achieving synergistic effects in chemokinetics/immune/hunger therapies [367]. Senfeng Zhao et al. designed Co4N/C nanocatalysts (Co4N/C NEs) based on LOX mimics to catalyze lactate oxidation. The nanocatalysts reversed the high lactate concentration in the TME and relieved its immune suppression, showing strong inhibitory effects on subcutaneous tumors and metastatic lymphoma [368]. In addition to enzyme mimics, some bacteria can also target the TME and consume lactate, working synergistically with nanodrugs to exert a good therapeutic effect. Tianqiu Xie et al. combined drug-loaded nanoparticles (DMnSH) with Vibrio elegans (Vei) to construct a probiotic-nanodrug conjugate, Vei@DMnSH. Vei@DMnSH accumulates at hypoxic tumor sites, consumes lactate and cysteine to reverse lactate-related immune suppression, and inhibits the biosynthesis of GSH, showing strong hypoxia-targeting and antitumor abilities [369].

### 4.3. Nanomedicine-Targeted Tumor Lipid Metabolism

Tumor cells reprogram lipid metabolism to provide sufficient energy for themselves, and the lipid metabolism reprogramming in the TME leads to a decrease in immune resistance against tumors, causing immune evasion. For tumors, such as glioblastoma, the microenvironment consists of cells with different cycling speeds. Fast-cycling cells exhibit mitochondrial dysfunction and primarily rely on glycolysis, while slow-cycling cells show increased lipid content, with lipids specifically metabolized under glucose deprivation conditions [370]. Due to the diversity of lipid types, their metabolic reprogramming is more complex [371]. Theoretically, any gene, receptor, enzyme, or signaling pathway related to lipid metabolism can be targeted; however, the differing lipid demands of various cells in the TME pose significant challenges to metabolic targeting in the TME [372]. Since nanomedicines exhibit better TME targeting properties, their mediation of lipid metabolism targeting offers distinct advantages.

#### 4.3.1. Nanoparticle-Based Drugs for Targeting Lipid Uptake, Synthesis, and Storage

The use of nanomedicines to inhibit the uptake of FAs, cholesterol, and other substances by tumor cells or immune cells in the TME is a promising approach for cancer therapy. Ezetimibe reduces cholesterol uptake by inhibiting the expression of the cholesterol transport protein NPC1L1 in the small intestine. Tarek A. Ahmed and colleagues utilized D-α-tocopheryl polyethylene glycol succinate (TPGS) and Pluronic co-polymers to deliver Ezetimibe via mixed micelles, significantly inhibiting tumor cells [373]. Jia Ma et al. developed a composite hydrogel platform (iFCuS-M/SSO@Gel), loaded with ferroptosis suppressor protein 1 (iFSP1) inhibitor and CD36 inhibitor (SSO). This platform targets CD36 on tumor-resident immune cells (TRICs), inhibiting lipid uptake and alleviating their immunosuppressive phenotype, thereby promoting CD8+ T cell infiltration. iFCuS-M/SSO@Gel enhances immunogenic cell death (ICD) through LPO, effectively inhibiting tumor growth, recurrence, and metastasis, demonstrating significant therapeutic efficacy [374].

Targeting and interfering with lipid synthesis pathways using nanoparticle-based drug delivery can effectively disrupt endogenous lipid metabolism and utilize lipid depletion strategies to kill tumors. Arif Khan et al. developed DSPC/Chol and DOPE/CHEMS immunoliposomes, which were conjugated with anti-Her-2 Fab’ and encapsulated FASN siRNA targeting breast cancer cells. Through the targeted delivery of FASN siRNA, they significantly inhibited FASN expression, thereby suppressing the proliferation of breast cancer cells [375]. Advait Shetty et al. constructed a unique paclitaxel–poly(lactic-co-glycolic acid) (PLGA) nanoparticle (PPNP) formulation, using it to enhance the delivery of gemcitabine. Following drug administration, key enzymes involved in fatty acid synthesis, including ACC, FASN, Lipin, and ATP-citrate lyase, were all inhibited, significantly disrupting the lipid synthesis pathway and inducing apoptosis in tumor cells [376]. Khan A et al. developed FASN siRNA-Encapsulated Her-2 Targeted Fab’-Immunoliposomes, which enhanced drug targeting for Her-2 positive breast cancer cells, and inhibited tumor cell survival by silencing FASN expression [377]. Shaimaa M Badr-Eldin et al. constructed FLV-MEL nanoconjugates for the combined therapy of fluvastatin (FLV) and melittin (MEL), which, by inhibiting HMG-CoA reductase, reduced cholesterol synthesis and cooperatively killed ovarian cancer cells with MEL [378].

Nanoparticle-based drugs can be used to disrupt lipid storage, consume key components, and interfere with key pathways in tumor cell lipid metabolism, ultimately impairing tumor cell function. Wenyao Zhen et al. constructed a COD-functionalized nanometal-organic framework, Hf-TBP/COD, utilizing cholesterol oxidase (COD) to enhance cholesterol consumption, increase tumor cell membrane tension, and ultimately disrupt the mechanical properties of the lipid bilayer. This led to a significant decrease in tumor cell migration energy and an increased tendency for rupture mediated by caspase-1-dependent pyroptosis, along with a reduced tolerance to oxidative stress [379]. Jialing Guo et al. constructed a biomimetic “dual-enzyme catalyzed cascade nanoreactor” (DOX@COD-MOF@CCM), which uses COD to deplete cholesterol content in cancer cells, reducing the stiffness of cancer cell membranes and restoring drug sensitivity to DOX in resistant cells, achieving a 94.4% tumor growth inhibition [380]. Jie Zhou developed a series of homologous multivalent supramolecular nanoparticle traps derived from heptakis (2,3,6-tri-O-methyl)-β-cyclodextrin (TMCD), where the nanoparticle trap PTMCD4 demonstrated the most powerful cholesterol capture and consumption capacity. This reduced tumor cell cholesterol and phosphatidylcholine levels, disrupted lipid metabolic homeostasis, and activated lysosomal stress, significantly inducing pyroptosis in a portion of the tumor cells [381].

In addition to inhibiting the normal functioning of lipid metabolism pathways to reduce lipid intake and consumption, the overexpression of lipid intake-related proteins can also be utilized to enhance the targeting ability of nanoparticle-based drugs. The SR-B1 receptor is highly expressed in malignant breast tumors in most patients [382]. Based on this phenomenon, Rebecca Johnson et al. constructed recombinant high-density lipoprotein (rHDL) nanoparticles as a drug delivery system for triple-negative breast cancer. rHDL NPs specifically interact with the SR-B1 receptor, significantly promoting drug uptake by tumor cells, thereby achieving targeted delivery through tumor cell lipid metabolism-related targets [383]. Yongfang Zheng et al. incorporated the peptide ligand (Pep2) of the CD36 receptor into doxorubicin-loaded DSPE-PEG_2000_ micelles, enhancing the receptor specificity of the drug-loaded nanomicelles and increasing the affinity of the nanoparticle drug for CD36-positive cell lines [384].

#### 4.3.2. Nanomedicine for Targeted Modulation of Endogenous Lipid Oxidation

FAs are crucial energy sources, and the metabolic reprogramming of FAO in the TME is a key driver of cancer progression. CPT1 is considered a rate-limiting enzyme in FAO and can serve as a pharmacological target [385]. Asia Saorin et al. developed nanomedicines based on the CPT1A inhibitor amiodarone, achieving liposomes and amiodarone particles with high drug loading, which exhibited significant efficacy against apoptosis-resistant cells [386]. Raffaele Conte synthesized biodegradable polyethyleneimine-functionalized polyhydroxybutyrate nanoparticles (PHB-PEI NPs) through aminolysis for the delivery of microRNA-124 (miR-124), which inhibits CPT1A expression and interferes with the functionality of malignant tumor cells [387]. West Kristian D. Paraiso et al. employed poly-ion complex (PIC) micelles to deliver CPT1A inhibitors (±)-, (+)-, and (−)-C75-CoA-C75 (4-methylene-2-octyl-5-oxo-tetrahydrofuran-3-carboxylic acid), achieving FAO inhibition and significantly reducing ATP levels. This strategy effectively enhanced drug penetration in 3D spheroid models and provided a novel approach for the delivery of CPT1A inhibitors in brain tumors [388].

### 4.4. Nanoparticle-Based Drug Targeting of Tumor Amino Acid Metabolism

Amino acid metabolism undergoes reprogramming in tumors, and the traditional definitions of EAAs and NEAAs do not apply to cancer cells. Targeting specific amino acid metabolism in the TME is an emerging approach for cancer therapy [192]. As mentioned earlier, the microenvironment contains many conditional amino acids, but there are still limited developments in nanomedicine targeting these metabolites. The conditional amino acids in the TME that are most commonly targeted by nanomedicines include glutamine, tryptophan, and arginine.

Gln in cancer cells serves as a nitrogen source for nucleotide biosynthesis and is a critical backup energy source for tumor cells. Nanomedicines can target Gln transporters to reduce Gln uptake by tumor cells, disrupting their nucleotide metabolism. Linping Zhao and colleagues developed a drug co-delivery system based on Ce6 (chlorin e6) and V9302 through self-assembly technology, named CeV. CeV preferentially accumulates in the TME via the EPR effect. Subsequently, V9302-mediated the inhibition of the type 2 alanine-serine-cysteine transporter (ASCT2), and reduces Gln uptake, successfully enhancing PDT-induced oxidative damage to tumor cells [389]. The team further extended this approach by constructing carrier-free self-assembled nanoparticles containing Ce6, ASCT2 inhibitor V9302, and PD1/PDL1 checkpoint blockade agent (BMS-1). This formulation inhibits Gln metabolism while upregulating Fas expression to enhance immune cell recognition of the tumor and blocking PD1/PDL1 to reduce immune evasion, achieving multimodal synergistic tumor treatment [390]. Targeting key enzymes in the Gln metabolism pathway to disrupt metabolic circuits can reshape the TME and induce tumor suppression. GLS1 catalyzes the conversion of Gln to glutamate, which is crucial for nitrogen metabolism, energy metabolism, and redox balance in cells [391]. Yiwei Dai et al. used Pluronic F127-stabilized Fe-DBEF nanoparticles loaded with GLS1 inhibitor BPTES and DOX. TME -triggered acidification caused the release of drugs from Fe-DBEF nanoparticles, disrupting Gln metabolism and increasing ROS levels, thereby synergistically enhancing chemotherapeutic and chemotherapy-induced oxidative damage [392]. Yingying Xu and colleagues employed Mg-Al LDH nanosheets to deliver siGLS1, reducing GLS1 expression. This led to inhibited cancer cell proliferation and effectively suppressed the growth of xenograft models [393].

Abnormal tryptophan metabolism in the TME is closely linked to immune suppression. The inhibition of tryptophan metabolism pathways can eliminate immune suppression within the microenvironment [394]. IDO1 is the rate-limiting enzyme in tryptophan catabolism, and the overexpression of IDO1 in many tumors reduces Trp levels and produces toxic kynurenine (Kyn) metabolites that suppress immune cells. Inhibiting IDO1 has emerged as a promising strategy for cancer therapy [395]. Xiuying Duan and colleagues synthesized nanoparticles loaded with IDO1 inhibitor NLG919 and STING agonist MSA-2. These nanoparticles efficiently accumulated in the tumor and significantly increased the bioavailability of NLG919, downregulating the conversion of Trp to Kyn, reshaping the immune-suppressive TME, and activating the STING pathway to enhance immune responses, ultimately inhibiting melanoma development [396]. Mengna Wang and colleagues developed HMP1G nanoparticles carrying IDO1 inhibitor 1-methyltryptophan (1-MT) and S-nitrosoglutathione (GSNO), a nitric oxide donor. HMP1G nanoparticles responded to the TME and released drugs, generating ROS and nitric oxide that synergistically inhibited IDO1 and reduced kynurenine accumulation in tumors, alleviating immune suppression caused by kynurenine/tryptophan metabolism [397].

In normal cells, Arg is synthesized from citrulline and aspartate via ASS1 and argininosuccinate lyase (ASL). However, in more than 70% of tumors, the transcription of ASS1 is suppressed, leading to an increased external demand for arginine, which results in elevated Arg consumption. Thus, Arg deprivation has become a potential strategy in immunotherapy [398]. However, Arg deprivation presents a double-edged sword. Immune cells also have a significant demand for Arg and depriving it may suppress immune cells in the TME, thus diminishing the antitumor effect. Therefore, targeting or modulating the Arg metabolic pathway in isolation is challenging for effective tumor therapy [399]. Junlei Zhang and colleagues cleverly addressed this issue by utilizing multivesicular liposomes (MVLs) to deliver L-arginine, ensuring its continuous supply to immune cells, while using CAT-2 shRNA to downregulate CAT-2 expression in melanoma cells. This dual approach successfully activated immune cells and partially weakened Arg uptake by tumor cells, leading to tumor suppression [400].

### 4.5. Nanoparticle-Based Drug Targeting of Tumor Nucleotide Metabolism

Nucleotide metabolism is divided into two important pathways: de novo nucleotide synthesis and nucleotide salvage. Research indicates that inhibiting de novo nucleotide synthesis does not cause significant toxicity to normal cells in the body, as it seems that most cells in terminally differentiated tissues can generate sufficient (d)NTPs via the salvage pathway to maintain homeostasis [287]. However, tumor cells, with their extensive replication demands, require ample genetic material, which cannot be maintained through a single pathway. Therefore, abnormal nucleotide metabolism is commonly found in the TME. In addition to inhibiting the intake of aspartate and glutamine, which are precursors of nucleotide metabolism, to treat tumors, inhibiting key enzymes in the nucleotide metabolism pathway also plays a crucial role. Many clinical drugs are designed based on their impact on the nucleotide metabolism pathway to achieve therapeutic outcomes. Current nucleotide metabolism inhibitor targets include DHODH, inosine monophosphate dehydrogenase 1 and 2 (IMPDH 1/2), thymidylate synthase (TS), dihydrofolate reductase (DHFR), RNR, glycinamide ribonucleotide transformylase (GART), and 5-amino-4-imidazolecarboxamide ribonucleotide transformylase/IMP cyclohydrolase (ATIC), among others. However, drug delivery efficiency remains a major limiting factor. The use of nanodrugs can effectively target the TME, enhancing drug efficacy.

DHODH is a key enzyme in the pyrimidine biosynthesis pathway, primarily responsible for catalyzing the conversion of dihydroorotate to orotate, a central reaction in pyrimidine biosynthesis. The inhibition of DHODH activity promotes tumor apoptosis. Leflunomide (LEF) inhibits DHODH and has recently been found to exhibit excellent antitumor effects, although it is associated with significant toxic side effects. To address this issue, Mariam Zewaila et al. developed cubosomes via an emulsification method, enhancing the delivery efficiency of LF and reducing the drug’s toxic side effects [401]. Teriflunomide (TFM), the active metabolite of Leflunomide, exerts the same mechanism of action by inhibiting the conversion of dihydroorotate to orotate, thereby disrupting the de novo pyrimidine biosynthesis pathway in tumor cells. Studies have shown that TFM demonstrates good efficacy against brain tumors; however, oral administration carries a high risk of hepatotoxicity. Dnyandev Gadhave developed TFM-loaded nano lipid-based carbopol-gellan gum in situ gel (TNLCGHG), which is delivered nasally and effectively reaches the brain to treat glioma, with no harmful effects on normal cells [402].

TS is a critical enzyme in thymidylate (TMP) synthesis, converting deoxyuridine monophosphate (dUMP) to deoxythymidine monophosphate (dTMP), thus providing thymine for DNA synthesis. Due to the essential role of thymidylate in DNA synthesis, TS is a key target in anticancer therapy. 5-fluorouracil (5-FU), a pyrimidine analog, inhibits the biological activity of TS and is one of the broad-spectrum anticancer drugs used in the treatment of malignant tumors, such as breast cancer. However, 5-FU has a short half-life, widespread distribution, and causes various side effects [403]. Sadia Anjum et al. synthesized thiolated chitosan-based nanoparticles for the targeted delivery of 5-FU. These nanoparticles, coated with HA, actively target CD44 receptors, which are highly expressed in solid tumors, and the constructed nanoparticles exhibited significant tumor cell cytotoxicity [404]. Jianping Liu et al. developed nanoparticles based on copper-doped layered double hydroxide (Cu-LDH) for the co-delivery of 5-FU and albumin-bound paclitaxel (nAb-PTX). The 5-FU/Cu-LDH@nAb-PTX nanodrug exhibited excellent TME targeting and sensitivity to low pH and laser, enabling controlled drug release and the efficient synergistic treatment of tumors in the TME [405].

RNR is a core catalytic enzyme in DNA synthesis, responsible for reducing ribonucleotides (such as ADP, GDP, CDP, UDP) into deoxyribonucleotides (dADP, dGDP, dCDP, dUDP). Hydroxyurea (HU) inhibits RNR, reducing the production of deoxyribonucleotides, which limits the supply of DNA synthesis substrates and thus affects the division and proliferation of tumor cells. However, HU can lead to side effects, such as rashes, leg ulcers, and leukopenia, during treatment [406]. Azam Akbari et al. utilized liposomes as drug delivery carriers to deliver Hydroxyurea, enhancing its cytotoxicity against tumor cells [407]. Fateme Azemati et al. constructed magnetic iron oxide nanoparticles (Fe_3_O_4_ NPs) containing Hydroxyurea, using an external magnetic field for nanoparticle-assisted delivery and employing magnetic resonance imaging to track these nanoparticles. Meanwhile, the superparamagnetic nanoparticles generate heat under the influence of the external magnetic field. This nanodrug enables targeted delivery of HU and can be used for synergistic tumor therapy [408]. Yerkeblan Tazhbayev et al. utilized human serum albumin nanoparticles (HSA-NPs) for HU delivery, ensuring that HU retains its chemical properties without being altered by fixation in the polymer matrix. The delivery system successfully extended the drug’s action time [409].

Methotrexate (MTX) and Pemetrexed (PMX) are both folic acid analogs that competitively inhibit various enzymes involved in folate metabolism, such as DHFR, TS, ATIC, and GART. The development of MTX and PMX as multitarget inhibitors holds great promise for optimizing cancer therapy. MTX can interfere with one-carbon metabolism and de novo purine and pyrimidine synthesis, thereby blocking the production of DNA and RNA and impacting tumor nucleotide metabolism. Xinye Wang et al. synthesized nanoparticles assembled from ferrous ions and histidine to deliver MTX to tumor sites, enhancing the sensitivity of tumor cells to MTX through histidine metabolism. MTX and its derivatives, such as MTX polyglutamate, inhibit the activity of key metabolic enzymes, including DHFR, ATIC, and TS, thereby disrupting one-carbon metabolism and de novo purine and pyrimidine synthesis. Concurrently, histidine metabolism depletes intracellular THF, further enhancing the inhibition of one-carbon metabolism [410]. Jiko Raut et al. conjugated MTX with ZnO quantum dots functionalized with amines (diethylenetriamine), and the delivery system interacted with MTX through noncovalent interactions. When the pH is less than 5.5, the ZnO QDs rapidly dissolve into Zn, significantly enhancing drug release efficiency and exhibiting potential microenvironment-responsive properties [411]. Juan Chen et al. constructed tMTX-PMX-PCNPs using PEGylated CNPs to co-deliver MTX and PMX. The co-delivery nanoparticles (MTX-PMX-PCNPs) demonstrated good dispersion and sustained-release behavior, showing potent antitumor efficacy in a mouse lung cancer model [412]. Mohammad Mohajeri et al. combined PEG-modified PMX (PEG-Cit-PMX), poly(N-isopropylacrylamide) (PNIPAM), PEG-thiol (PEG-SH), and gold nanoparticles (GNPs) to construct a composite nanoplatform. PEG-Cit-PMX specifically binds to folate receptors for the active targeting of cancer cells, accumulates in the TME, exerts antitumor effects via GNPs and PMX, and enhances CT imaging. Thus, a combined imaging and therapeutic antitumor nanodrug was developed [413].

### 4.6. Nanomedicine-Mediated Targeting of Redox Metabolism and Ferroptosis

GSH is a tripeptide composed of glutamic acid, cysteine, and glycine, which is often highly expressed in the TME. As an important antioxidant, it participates in redox reactions, scavenges free radicals, maintains redox homeostasis, and protects cells from damage caused by ROS. Therefore, disrupting the metabolic stability of GSH in the TME, thereby inducing oxidative stress in tumor cells, has become a significant strategy in cancer treatment. Currently, two main approaches to disrupt GSH metabolism are the following: depleting GSH or inhibiting Glutamate–Cysteine Ligase (GCL). However, the development of nanoparticle-based drugs targeting GCL is still in its early stages. Currently, most nanoparticle drug research focuses on the depletion of GSH.

Depleting GSH effectively inhibits the antioxidant capacity of tumors, thereby enhancing therapeutic efficacy. Lianhua Fu et al. synthesized degradable copper-doped calcium phosphate nanoparticles (CuCaP NPs), termed PGC-DOX nanoplatforms, by using polyethylene glycol (PEG)-modified GOx as a template. The PGC-DOX platform is capable of delivering DOX. After releasing the drug in response to the low pH of the TME, Cu ions significantly deplete GSH, achieving GSH depletion and thus enhancing the efficacy of Chemodynamic Therapy [414]. Furthermore, a DTX@PAMBE-SS-TPGS nanoparticle, composed of polymers containing disulfide and ester linkages along with d-α-tocopherol polyethylene glycol succinate (TPGS), was constructed to load docetaxel (DTX). The DTX@PAMBE-SS-TPGS nanoparticles are delivered to the TME through the enhanced EPR effect. The disulfide and ester bonds in the nanoparticles respond to the high concentration of GSH in tumor cells, depleting GSH and elevating ROS levels. This results in the release of the encapsulated DTX, disrupting the redox balance within tumor cells, inducing oxidative stress, and enhancing the sensitivity of the tumor to DTX [415].

In addition to the redox homeostasis associated with GSH, the disruption of lipid-related redox homeostasis leading to ferroptosis has also become an important direction in cancer therapy. Many cancer cells exhibit increased susceptibility to ferroptosis, and inducing ferroptosis can be explored as an anticancer therapy. Ziting Zhang et al. utilized biomimetic nanoscale carriers RSV-NPs@RBCm to deliver the important Traditional Chinese Medicine (TCM) chemical monomer resveratrol (RSV). RSV induced an increase in ROS and lipid peroxidation in colorectal cancer cells, leading to ferroptosis and subsequent cell death [416]. LOX is a key driver of ferroptosis, and the unsaturation of lipid bilayers is a critical factor determining cellular sensitivity to ferroptosis. Targeting LOX has become a promising direction for inducing ferroptosis in tumor cells [417]. Zhengcong Cao et al. identified the key LOX ALOX15 and used Angiopep-2-modified macrophage membranes (MMs) to encapsulate a small activating RNA (saALOX15) targeting ALOX15 in mesoporous polydopamine (MPDA) nanoparticles. This approach significantly enhanced the drug’s ability to penetrate the blood–brain barrier, disrupt the glioblastoma cell membrane, and induce ferroptosis in these cells [418].

## 5. Applications and Challenges of AI in Nanodrug Design, Evaluation, and Precision Therapy

**Figure 4:**
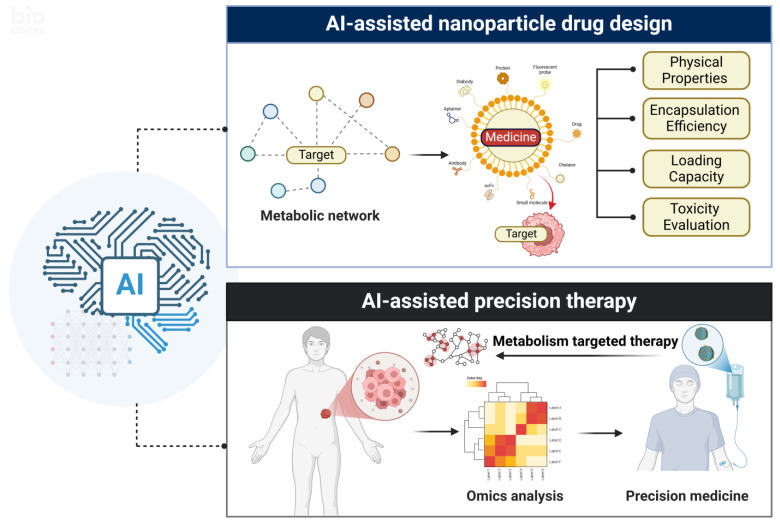
Artificial Intelligence (AI)-assisted nanoparticle drug development and individualized medication throughout the entire process. AI can integrate metabolomics data to identify key targets in tumor metabolic pathways for subsequent research and development. In the future, AI, through nanoparticle material-related databases, can provide the most precise predictions for the physicochemical properties, encapsulation rate, drug loading capacity, toxicity, and interactions with the tumor microenvironment, eliminating the trial-and-error process of traditional drug development and maximizing benefits. AI can also assist in personalized multiomics analysis, offering optimal medication recommendations and prognostic feedback based on the patient’s individualized information, preventing patients from receiving suboptimal results due to heterogeneity. Created with Biorender.com (accessed on 24 February 2025).

AI technology refers to the use of machines to perform tasks that were originally only possible for humans to accomplish. With the development of technology, AI has demonstrated potential that exceeds that of domain experts in many areas, especially when working with large datasets to uncover deep information that is difficult for humans to detect. The development of nanomedicines and drug carriers involves vast amounts of data, such as molecular structures, biomarkers, and clinical manifestations, which is where AI can play a significant role. Furthermore, precision therapy is a key application of nanomedicine delivery technology. Al can enable precise diagnosis, providing accurate guidance for subsequent treatment plans for patients [419,420]. AI can also assist in target screening based on metabolomics, enabling precision therapy (Figure 4) [421].

### 5.1. An Overview of Commonly Used Artificial Intelligence Models

SVM and SVR: Support Vector Machine (SVM) is a supervised learning algorithm widely used in classification. Support Vector Regression (SVR) is a method where SVM is used for regression tasks. SVM and SVR are particularly proficient in handling high-dimensional data. Its core idea is to construct a hyperplane that separates data points of different classes and maximizes the margin between the hyperplane and the data points, thereby achieving optimal classification [422].

Decision Tree and Random Forest: Decision Tree is a model based on a tree structure, primarily used for classification and regression tasks. It classifies or predicts input data through a series of decision rules [423]. Each node of the tree represents a test of a feature, each branch represents the outcome of that feature’s test, and the leaf nodes represent the final classification or predicted value. Random Forest is a method in Ensemble Learning based on the concept of Decision Trees. It improves the performance and robustness of the model by constructing multiple Decision Trees and aggregating their prediction results. Specifically, a random forest consists of several Decision Trees, which are randomized during training (such as the random selection of data subsets and features), and then the final prediction is obtained through voting (for classification) or averaging (for regression) [424].

Gaussian Process: the Gaussian Process is a nonparametric method, which means it does not rely on a predefined fixed parametric function form. Instead, it directly infers the distribution of the function from the training data. By selecting appropriate kernel functions and hyperparameters, the Gaussian Process can flexibly model complex functional relationships [425].

K-means: K-means is a common unsupervised learning algorithm, primarily used for clustering analysis. Its goal is to partition the samples in a dataset into K clusters, such that data points within the same cluster are as similar as possible, while data points in different clusters are as distinct as possible [426]. The K-means algorithm performs clustering by minimizing the squared error of data points within each cluster. The main advantage of this unsupervised learning algorithm is that it could be easily applied to large, difficult-to-manually-label datasets, making it more convenient compared to supervised learning models.

ANN: an Artificial Neural Network (ANN) is a computational model inspired by biological neural networks, designed to simulate the way the human brain processes information [427]. The network consists of an input layer, an output layer, and several hidden layers. Each layer contains many neurons, and neurons between adjacent layers are connected with variable weights. The weighted results are then passed through a nonlinear activation function. Neural networks have various variants, such as the Recurrent Neural Network (RNN), Convolutional Neural Network (CNN), and Graph Convolutional Network (GCN) [428,429,430].

### 5.2. AI in Nanodrug Design

Nano delivery systems require the careful design of targets, carrier size, encapsulation capacity, drug loading capacity, binding affinity, toxicity evaluation, and delivery efficiency to ensure the proper matching and stability of nanodrugs. AI can empower the above research directions and provide assistance throughout the entire process of nanomedicine design.

#### 5.2.1. Screening Key Targets in Tumor Metabolic Pathways

Metabolomics can identify cancer biomarkers and driving factors of tumorigenesis by analyzing key metabolites in biological fluids, cells, and tissues to discover biomarkers of tumors [431]. For example, David A. Gaul and colleagues utilized a SVM model to process and analyze large-scale metabolic data, significantly improving the prediction accuracy and sensitivity. Their study identified 16 metabolites as potential biomarkers for ovarian cancer, and these biomarkers were able to distinguish cancerous samples from normal samples with 100% accuracy [432]. The reprogramming of tumor-associated metabolic pathways is highly complex and extensive. Traditional drug screening methods are still inefficient and time-consuming. Interactions between metabolites are complex and often involve nonlinear relationships. DL models introduce nonlinear activation functions between neurons in each hidden layer, which often gives them an advantage over traditional ML models. They have significant advantages in big data processing, enabling a more efficient identification of critical targets in tumor metabolism [433]. Fadhl M Alakwaa and colleagues applied a Feedforward Network (FFN) to select important metabolites and used them to complete the binary classification task of ER-positive and ER-negative. Since DL has the highest accuracy among seven algorithms, the metabolites it selects are more valuable for reference. The study examined 25 activation nodes in the first hidden layer, selecting metabolites with absolute weight values greater than 0.1, and then conducted pathway analysis using the relevant dataset. It is worth noting that the interpretability of DL models is relatively poor, and it is difficult to fully understand the meaning of each node in the entire model. The approach used in the above study, which only selects the first-layer neurons, may still overlook information from deeper layers [434].

#### 5.2.2. Carrier Size

One advantage of nanocarriers is that their size can be adjusted. The size of nanoparticles used in drug delivery systems should be small enough to avoid being captured by macrophages in the liver and spleen, but also large enough to prevent rapid leakage into the bloodstream [435,436,437]. The fenestration size in the spleen’s sinusoids and the pores in the Kupffer cells of the liver range from 150 to 200 nanometers, while the gaps between endothelial cells in tumor blood vessels may range from 100 to 600 nanometers [435]. Therefore, the size of nanoparticles used for drug delivery should be controlled to be no larger than 100 nanometers to achieve the ideal effect. In one study, researchers led by Gokay Yamankurt optimized the size and surface modification of nanoparticles through high-throughput immunogenicity screening and ML algorithms, such as linear regression and logistic regression, to achieve the optimal design for specific sizes and shapes [438]. Additionally, Flore Mekki-Berrada and colleagues used a combination of Gaussian processes and deep neural networks (DNNs) to quickly converge and optimize the size and shape of nanoparticles with predefined absorption spectra based on a small amount of experimental data [439]. Tell Tuttle et al. combined molecular dynamics simulations with ML techniques to develop a high-throughput screening platform based on a coarse-grained force field, aimed at exploring the size characteristics of nanoparticles formed from various chemical structure combinations. This method rapidly calculated 8,000 possible tripeptide combinations in the chemical space and screened candidates capable of self-assembling into specific-sized nanostructures. This approach revealed the self-assembly rules of peptides and provided guidance for the design of peptide-based nanodrugs [440]. These AI-driven approaches significantly improved the efficiency of nanocarrier design and provided the data support and theoretical foundation for predicting their biological behavior.

#### 5.2.3. Carrier Encapsulation Efficiency

Encapsulation efficiency is also an important metric that needs to be considered in nanomedicine design. Omar M Fahmy et al. used CatBoost, linear regression, SVR, and ANN to predict the encapsulation efficiency in nanomaterials, such as niosomes. They developed models capable of mining previous research data to predict important properties, such as the encapsulation efficiency of specific types of nanoparticles. Additionally, they emphasized the classification features to improve the robustness of the algorithms. The study concluded that the CatBoost algorithm performed the best. The results of the CatBoost model revealed that the lipid ratio was the main factor influencing the encapsulation efficiency, followed by the molar ratio of the lipid surfactant. The model can be used to assist in improving drug encapsulation efficiency, minimizing experimental procedures and costs [441]. Weibin Liang et al. optimized the synthesis of enzyme/metal-organic frameworks using ML models, such as random forests, achieving an increase in the overall performance index (OPI) by at least 1.3 times compared to systematic seed data studies. The model includes 84 seed data points for glucose oxidase and horseradish peroxidase, optimizing the synthesis formulation process and eliminating the subjectivity introduced by human reliance on experience and intuition [442].

#### 5.2.4. Drug Loading Capacity

The drug loading capacity of nanomedicines is closely related to their ultimate therapeutic efficacy [443]. Rania M. Hathout et al. predicted the drug loading capacity in solid lipid nanoparticles (SLNs) by performing molecular docking experiments on a tripalmitin matrix simulated using the GROMACS software package. Using supervised ML techniques, such as Gaussian processes, they correlated drug descriptors (such as molecular weight (M.W.), xLogP, TPSA, and fragment complexity) with molecular docking binding energies. By inputting descriptors as variables, they accurately estimated the drug loading in the studied solid lipid nanoparticles. The model can predict and optimize the drug loading capacity in solid lipid nanoparticles, providing initial and accurate predictions of drug–carrier interactions before experimental wet-lab work, which helps optimize drug–carrier pairings and improves both in vitro and in vivo stability [444]. This research team also used ANN combined with molecular dynamics simulations to predict the binding energy (ΔG) between drugs and polymer carriers (such as tripalmitin and PLGA), achieving high-precision predictions of drug loading capacity, with a correlation of R^2^ = 0.9. These studies demonstrate the powerful potential of AI in optimizing drug loading capacity [445].

#### 5.2.5. Binding Affinity Determination

Nanomedicines provide a tool for personalized targeting by coating the surface of drug-loaded nanoparticles with specific ligands (such as antibodies, membrane-binding receptor ligands, and other cell markers) or using natural cell membrane vesicles. These particles selectively bind to target cells near the target tissue, reducing off-target effects and related side effects [446]. Therefore, it is crucial to design nanodrugs with targeting capabilities. The interaction of nanodrug properties with plasma, vascular endothelium, and cell membranes is not easy to rationalize, but it can be significantly improved using computational methods. The use of traditional ML tools in research is relatively limited; computational methods are employed to calculate binding affinity. For example, the Metropolis Monte Carlo algorithm can be used to calculate the optimal antibody surface coverage for nanoparticles binding to specific vascular endothelium [447]. Changying Shi and colleagues designed nanocarriers by customizing linear dendritic copolymers (telodendrimers) to enhance drug delivery efficacy. The research team employed computational virtual screening methods, combining small molecule libraries and peptide chemistry, to optimize the structure of the nanocarriers, ensuring both drug-binding affinity and carrier stability. Additionally, molecular dynamics and Monte Carlo simulations were used to predict and validate the interactions between the drug and the nanocarrier, aiming to improve drug targeting, controlled release, and anticancer efficacy in tumors. This method expands the chemical space through virtual screening, enabling the discovery of chemical combinations that are difficult to explore through traditional empirical methods and quickly identifying efficient candidate molecules [448]. With the development of DL technology, models like CNN have been applied to predict drug-target binding affinity. Qichang Zhao et al. proposed AttentionDTA, which achieved excellent performance on the drug-target affinity (DTA) dataset. While CNNs are typically used for processing two-dimensional or three-dimensional information, the convolution process is also effective when applied to one-dimensional sequence data, as it preserves local features while reducing sequence length to improve efficiency. The researchers used one-dimensional CNN to process the SMILES strings of drugs and the amino acid sequences of proteins to predict binding affinity, and incorporated an attention mechanism to focus on important subsequences, enhancing the model’s interpretability [449].

#### 5.2.6. Toxicity Evaluation

When using innovative nanomaterials for drug development, it is essential to consider the potential toxicity and contamination of nanodrugs. Various methods exist in computational modeling, such as Nanoparticle Quantitative Structure-Activity Relationship (Nano-QSAR), analogy methods, and data-driven analysis. Nano-QSAR, nano-QNTR (where N represents nanostructures and T represents toxicity) and nano-QNAR (where N represents nanostructures and A represents activity) methods can be used to predict the potential toxicity of nanomaterials [450]. In inhalation nanotoxicology, mechanical dose models can effectively predict the deposition of nanoparticles. It is challenging to experimentally determine the dose that can be absorbed by the body, whether in humans and animals. Therefore, Multi-Path Particle Dose (MPPD), as a quantitative tool, can predict the deposition of inhaled nanoparticles in the lungs of humans and other mammals. The MPPD models developed by the National Council on Radiation Protection and Measurements (NCRP) and the International Commission on Radiological Protection (ICRP) can accurately predict the local and regional deposition and clearance of particulate matter in the lungs, providing essential theoretical foundations and tools for the inhalation dose prediction of nanoparticles [451,452].

Mei Liu et al. developed a ML model that combines the chemical, physical, and phenotypic characteristics of drugs to accurately predict potential adverse drug reactions (ADRs) and toxicity. This model analyzes existing data to assess the potential risks of drugs, offering early warning before the actual synthesis of the drug, significantly improving drug development efficiency and reducing the risk of failure in later clinical trials and safety issues. This research demonstrates the immense potential of ML technology in drug toxicity prediction and helps develop safer and more effective drugs [453]. Another example is the work by Mahnaz Ahmadi et al., who used ML models to predict the toxicity of nanoparticles. These models utilized the physicochemical properties of nanomaterials, such as particle size, surface charge, and biocompatibility parameters, along with cytotoxicity experimental data, to build toxicity prediction models. Algorithms, such as random forest, decision trees, and SVM, were employed to analyze the toxicity differences of nanocarriers in various cellular environments. The study ultimately found that random forest exhibited the highest accuracy [454]. Moreover, QSAR models using DNN combined with molecular descriptors can predict drug-induced liver injury (DILI) [455]. AI models can learn from large datasets, such as Tox21 and ToxCast data, and automatically extract key features, improving prediction accuracy and efficiency. These AI-enhanced models significantly reduce the need for experimental validation, accelerating the toxicity screening and drug design process while optimizing the compound screening range and success rate [456,457]. These models can rapidly predict the toxicity of nanocarriers with different chemical structures in specific cells, providing strong support for optimizing design and reducing experimental screening costs.

#### 5.2.7. Evaluating and Optimizing Nanomedicine Delivery Efficiency

The primary goal of nanodrugs is to enhance drug delivery efficiency by delivering active drugs to pathological sites without harming healthy organs or tissues [458,459]. Nano delivery systems play a critical role in the delivery of biological drugs, such as RNA, and the delivery efficiency directly affects whether the drug can effectively reach target cells and exert its therapeutic effect. To ensure the stability and function of RNA drugs, nano delivery systems must provide support in multiple aspects, including the protection of RNA molecules, targeting accuracy, and timely drug release. Therefore, designing a properly evaluated system to improve delivery efficiency and ensure targeting is crucial for the clinical application of RNA drugs [460,461].

Morag Rose Hunter et al. used ML algorithms to analyze data phenotype fingerprints to assess mRNA delivery efficiency. The team extracted key features from images to identify mechanisms related to enhanced delivery. Advanced image analysis algorithms (using Columbus software) were employed to extract features from the images (up to 850 features). Subsequently, various ML models, such as random forests and SVM, were applied to train from these features and to deeply analyze the imaging data, understanding the impact of different intracellular transport pathways on mRNA delivery. This enabled the redesign of lipid nanoparticles (LNPs) to target specific cellular uptake pathways, significantly enhancing mRNA delivery efficiency both in vitro and in vivo [462]. Weichun Chou et al. innovatively integrated AI with physiologically based pharmacokinetic (PBPK) models, using ML and DNN algorithms that predict key parameters of PBPK models. By integrating AI-based QSAR models with PBPK models, they predicted the delivery efficiency and biodistribution of different nanoparticles in mouse tumors. The AI-assisted PBPK model was able to quickly predict the tumor delivery efficiency of nanoparticles and reduced reliance on animal experimental data, providing an efficient screening tool for the development of nanodrugs [463].

The ability of nanodrugs to cross biological barriers has a significant impact on their distribution concentration in the body, which directly affects drug efficacy and safety [464]. In drug development and clinical application, drug biodistribution is significantly influenced by the structure and function of biological barriers. For example, the blood-brain barrier, cell membranes, and barriers in internal organs can significantly affect the absorption, distribution, metabolism, and excretion of drugs [465]. Therefore, an in-depth study of the mechanisms and influencing factors of drug passage through biological barriers is of great theoretical and practical significance for optimizing nanodrug design, improving therapeutic effects, and reducing side effects.

Mingsheng Zhu et al. used DL-based image segmentation technology to establish Nano-ISML, a new method for high-throughput quantitative tumor vascular permeability analysis, which reveals tumor vascular permeability heterogeneity. They employed U-Net, a CNN network, to perform image segmentation, outlining the edges of each object to be detected. The model takes fluorescence images of two channels (vessels and nanoparticles) as input, providing segmentation results and overlapping them to calculate nine important metrics, including vascular permeability. This system enabled the transition from qualitative to quantitative vascular permeability analysis, providing theoretical support and design principles for the development of personalized nanodrugs [466]. Zhen Gao et al. used a SVM model to analyze clinical phenotypes of drugs, including side effects and indications as features, and successfully predicted the ability of drugs to cross the blood–brain barrier. The study showed that combining drug chemical structure and clinical phenotype features significantly improved prediction accuracy and provided new perspectives for the discovery and repurposing of central nervous system drugs [467].

### 5.3. AI for Precision Therapy

Currently, AI technology has already played a key role in precision diagnosis and treatment. AI-assisted analysis and prescreening for patient stratification helps to more quickly and accurately identify target patients, thereby allowing for the development of specific treatment plans, medications, or targets. It also lays the foundation for the design of nanomedicines. For instance, in a study involving tumor samples from 48 patients, researchers used an algorithm based on random forest classifiers to identify and categorize mutated cancer-driving factors by training with historical data. The research team then constructed a drug–target interaction database to target these cancer-driving factors and automatically match the appropriate treatment drugs based on each patient’s mutation profile. This approach also offers new solutions for screening nanomedicines that specifically target metabolic molecular pathways [468].

It is easy to imagine that in the future, AI will help us accurately identify more key molecular regulators involved in cancer gene metabolic reprogramming, driving transformative innovations in targeted cancer therapy. Research has shown that tumor metabolic reprogramming is closely related to tumor progression and metastasis [469,470]. Using AI technology to identify nanomedicines targeting metabolism reprogramming-related targets holds great potential in the field of precision therapy. Yangzi Chen et al. used ML techniques to analyze the reprogrammed metabolic profile data of gastric cancer (GC) patients, not only achieving a preliminary screening of GC patients but also completing the classification of stage IA and stage IB. The reprogrammed metabolites selected by the model demonstrated better performance than the model based on the three existing clinical biomarkers [471]. Furthermore, the metabolic prognostic model in this study was able to provide precise prognosis for individual patients, guiding subsequent treatment. Sunan Cui et al. utilized the actuarial deep learning neural network (ADNN) architecture to perform prognostic prediction based on multiomics data on a stage III non-small cell lung cancer dataset. The model analyzed multiomics data, including radiomics and transcriptomics, and achieved better performance than traditional prognostic models [472]. This multiomics system analysis approach can greatly expand the diversity of data and uncover more precise therapeutic targets for individual patients. Similarly, other researchers have established metabolomics and transcriptomics system databases for triple-negative breast cancer (TNBC) and used ML techniques to distinguish between different subtypes in both omics. This study showed that N-acetyl-aspartyl-glutamate is an important tumor-promoting metabolite and could be a potential target for treating a specific subtype of TNBC [421]. Based on these studies, with the development of DL technologies, future research could incorporate radiomics data into multiomics analysis [473]. In clinical practice, imaging technologies, such as CT and MRI, can directly capture tumor lesions, providing noninvasive information about the tumor tissue itself. By using ML techniques, especially CNN, we can extract deep, hidden information from the images to carry out radiomics research [474,475]. The synergy between radiomics and multiomics analysis can enable a comprehensive decoding and characterization of the TME [476]. Therefore, combining radiomics with systematic analysis is also an important approach to enhance precision diagnosis and treatment.

These studies highlight the potential of applying ML techniques in metabolomics to identify metabolic reprogramming treatment targets. Overall, the combination of nanomedicine and metabolomics provides a precise diagnostic and treatment process: constructing multiomics databases, identifying metabolic reprogramming treatment targets for each tumor subtype, providing a precise diagnosis for patients in clinical practice, and customizing nanomedicines for patients. AI technology will empower each step of this process, driving nanomedicine to achieve precision diagnosis and treatment.

## 6. Summary and Future Perspectives

With the continuous advancement of research, our understanding of the metabolic reprogramming of tumor cells and the TME has deepened significantly. This growing knowledge has revealed that metabolic alterations within the TME play a crucial role in influencing tumor progression, with certain metabolic pathways driving key processes like cell proliferation, survival, and metastasis. As the TME is a highly dynamic and complex environment, its metabolic landscape is vastly different from that of normal tissues, offering a window for therapeutic intervention. The ability to manipulate specific metabolic pathways to hinder the survival of tumor cells or disrupt the supportive roles of the TME has generated significant interest in cancer research. In this context, drugs targeting specific metabolic pathways have gradually entered clinical trials, demonstrating their potential as viable cancer therapies. This shift in focus towards tumor metabolism has proven that drug development based on tumor metabolism holds broad prospects and advantages, providing an exciting new avenue for therapeutic strategies. As research in metabolism-related omics, such as metabolomics, and the pathology of the TME deepens, the identification of key targets in tumor metabolism may pave the way for novel therapeutic strategies that disrupt the TME, ultimately improving clinical outcomes.

The innovation of nanobiotechnology has further accelerated the development of new drug delivery systems. Nanoparticles, due to their unique properties, offer enhanced drug delivery capabilities. By utilizing the passive targeting ability of nanoparticles, which allows them to accumulate at tumor sites due to the leaky vasculature of tumors, and combining this with actively designed targeting mechanisms, drugs can be delivered more precisely to the TME. This targeted approach allows for a higher concentration of drugs at the tumor site, enabling more accurate targeting of tumor cells. As a result, the systemic toxicity and side effects commonly associated with conventional chemotherapy can be reduced, as the drugs are confined primarily to the tumor tissue, sparing normal healthy cells. These advanced nanodrug systems hold great promise for improving cancer treatment outcomes by maximizing the therapeutic window while minimizing harm to the patient.

Although nanomedicines have shown remarkable advantages in targeted drug delivery to the TME, there is still much work remaining. Most nanodrugs targeting specific metabolic pathways have not yet been developed and remain in the experimental stages, without full optimization or validation in clinical settings. However, the rapid development of AI in the field of biomedical research holds great promise for accelerating this process. AI, with its exceptional ability to integrate vast datasets and make precise predictions, can significantly streamline the drug development pipeline. Once a comprehensive database of nanomaterials and drug interactions is established, AI could predict potential druggable targets within metabolic pathways more efficiently, providing valuable insights into the design and optimization of novel nanodrugs. AI can assist in the integration of large-scale omics data, such as genomics, proteomics, and metabolomics, to identify new therapeutic targets and biomarkers that can be exploited for precision medicine. It can also help in the development of multieffect synergistic nanodrugs, such as those related to metabolism-based therapies, including photodynamic therapy (PDT), sonodynamic therapy (SDT), and photothermal therapy (PTT). These therapies use light, sound, or heat to activate the therapeutic effects of the drug, potentially enhancing the drug’s effectiveness and targeting capability, especially in conjunction with nanomaterials that can be engineered to respond to specific metabolic changes in tumor cells. Moreover, based on omics technologies, AI can assist in personalized tumor patient analysis, predict the drug delivery and targeting efficacy, and reduce the therapeutic failure due to patient heterogeneity, ultimately improving patient prognosis. AI-assisted drug development and clinical application will become a new paradigm in the development and application of nanodrugs targeting abnormal metabolic pathways in the TME.

## Data Availability

No new data were created or analyzed in this study.
